# A novel panel based on immune infiltration and tumor mutational burden for prognostic prediction in hepatocellular carcinoma

**DOI:** 10.18632/aging.202670

**Published:** 2021-03-10

**Authors:** Chan Xie, Hewei Wu, Tao Pan, Xingrong Zheng, Xiaoan Yang, Genglin Zhang, Yunwen Lian, Jiaxin Lin, Liang Peng

**Affiliations:** 1 Department of Infectious Diseases, The Third Affiliated Hospital of Sun Yat-sen University, Guangzhou, Guangdong Province, China; 2 Department of Interventional Radiology, The Third Affiliated Hospital of Sun Yat-sen University, Guangzhou, Guangdong Province, China

**Keywords:** tumor mutation burden, hepatocellular carcinoma, immune infiltration, neoplasm recurrence, vascular invasion

## Abstract

Tumor mutation burden (TMB) has been associated with prognosis in various malignancies, but it has yet to be elucidated in hepatocellular carcinoma (HCC). We aimed to investigate the prognostic effects of TMB and its relationship with immune infiltration through multiple databases and whole-exome sequencing, so as to establish a panel model capable of predicting prognosis. The results demonstrated that the prognosis of high TMB group was worse than that of low TMB group, with a cutoff TMB value of 4.9. Enrichment analysis demonstrated that differentially expressed genes were mainly related to T cell activation, cell membrane localization and matrix composition. Tumor immune infiltration analysis revealed the infiltrations of Th2, Th17, and Tgd were up-regulated in high TMB group, while those of Tr1, MAIT, and DC were up-regulated in low TMB group. TMB-Infiltration model fit well with the actual survival observation, with a C-index 0.785 (0.700-0.870), which verified in ICGC-LIRI-JP was 0.650 (0.553-0.747). Additionally, these screened immune genes performed well in predicting tumor vascular invasion with a C-index of 0.847 (0.778-0.916). Overall, these results indicated that patients with high mutation frequency of immune-related genes and high TMB were prone to have worse prognosis and relapse after radical treatment.

## INTRODUCTION

Hepatocellular carcinoma (HCC) is the third most common digestive cancer in global incidence and mortality, ranks the fourth highest among all tumors. Rates of both incidence and mortality in men are 2 to 3 times higher than women, and the mortality of HCC ranks second only to that of lung cancer among men [1]. Although the pace of increase in women has slowed from previous years, incidence in men has continued to grow at a rate of 2% to 3% annually for the past decade [2]. Main risk factors for HCC are chronic infection with hepatitis B virus (HBV) or hepatitis C virus (HCV), alcohol consumption, aflatoxin-contaminated foodstuffs, smoking, obesity and type 2 diabetes [3]. Many HCC patients were diagnosed with advanced stage because of mild symptoms in the early stages. Treatment of HCC mainly includes surgical resection, radiofrequency ablation, liver transplantation, radiotherapy, transcatheter arterial chemoembolization (TACE) and targeted therapy [4]. In the past few years, immune checkpoint inhibitors (ICIs) was considered to be one of the most promising treatments in cancer immunotherapy. Several clinical trials of programmed death-1/programmed death-ligand 1 (PD-1/PD-L1) inhibitors in HCC have been conducted to show patient benefit [5, 6]. Tumor mutation burden (TMB) is one of the popular biomarkers for predicting the efficacy of PD-1 inhibitors. Previously studies demonstrated a correlation between high TMB and clinical benefits of PD-1 inhibitors in lung cancer [7]. HCC has a higher tumor mutation load than the average for other solid tumors [8]. In immunotherapy for most tumors, overall survival (OS) was significantly longer in patients with high TMB than in those with low TMB, showing a median overall survival difference of approximately 40% [9]. In the 2019 guidelines for non-small cell lung cancer (NSCLC), a new item of TMB was added to identify lung cancer patients who were eligible for dual immunotherapy with " Nivolumab plus Ipilimumab" and "Nivolumab" single-drug immunotherapy [10, 11]. However, the role of TMB in immunotherapy of HCC remained unclear.

Immunotherapy is aimed to arouse and strengthen the body's immune system to kill the tumor cells through various methods [12]. In this case, the higher TMB, the more personality of the tumor is different from normal tissue, and the easier it is to be the target of immune cells, so the more likely it is theoretically to response to immunotherapy [13]. Surrounding tissues, immune cells, blood vessels and extracellular matrix together constitute the tumor microenvironment, which is the "fertile soil" to help the tumor expand and invade faster [14]. Immune cells involved in tumor development are diverse and highly heterogeneous. Complex and subtle relationship between different types of immune infiltrating cells and cell receptors often related to vascular invasion and tumor escape [15]. Previous studies have shown CD8+ T cells that express different levels of PD-1 are enriched in HCC tissues [16]. A study of 5,000 T-cell sequencing data has described the landscape of infiltrating T cells in HCC, showing that large numbers of dysfunctional, killing T cells and inhibiting T cells clustered in tumor tissues [17]. In addition, depleted T cells may further potentially evolve into inhibitory T cells.

Vascular invasion (VI) has been widely demonstrated to be closely associated with poor prognosis in patients with HCC after surgery resection [18, 19]. Even in tumors of same stages receiving the same treatment, the prognosis is still different [20], attributing it to the biological behavior of the tumor. Microvascular invasion (MVI) prior VI and VI has attracted more attention, which proved to be high risk factor for early recurrence of HCC after operation [21]. Previous studies have indicated that HCC with microvascular invasion are needed to be comprehensively evaluated for a wider ablation margin and should be considered as a candidate for liver transplantation more cautiously [20, 22]. However, even accepting radical resection, almost half of the patients relapsed within 3 years [23]. At present, few studies have been conducted on the relationship between VI and infiltrating immune cells, nor on the relationship between recurrence and TMB in HCC.

By analogy with other tumor clinical findings, TMB does play a potential role in HCC-related immunity. But there is still lack of evidences on the relationship between HCC and TMB currently. The aim of our study is to explore the differences of immune infiltrating cells at different TMB levels, identify related genes and construct prognostic panels. Additionally, we hope to further explore the potential role of TMB in vascular invasion and HCC recurrence.

## RESULTS

### Characteristics of included patients and landscape of mutation profiles in HCC

Detailed characteristics of patients in the low TMB and high TMB group were presented in Table 1. There was a significant difference in TMB level between the two independent sets (P < 0.01). After matching with propensity score, composition of pathology grade was basically similar (P = 0.63). As for gender and age, there was no paramount difference between the two groups (P=0.23, P=0.61). It can be seen that age > 60 and male had sufficient advantages in the proportion of population, especially in the high TMB group (67.57%, 72.97%). Notably, a considerable proportion of people in high TMB group were infected with the virus (40.54% vs 10.81%, including HBV and HCV infection), suggesting that influence of potential virus DNA integration. No significant differences could be found in alcohol consumption and family history of cancer in our study cohort (P = 0.80, P = 0.76). 72.97% of patients in the high TMB group were in stage III-IV of TNM staging, and accounted for the majority of patients in ACJJ-T staging (72.97%), compared to patients in low TMB group (67.57%).

**Table 1:** Clinical baseline of HCC patients screened in the TCGA cohort study.

**Characteristics**	**Low TMB, n=37(%)**	**High TMB, n=37(%)**	**P value**
**Age**			
**>60**	20(54.05%)	25(67.57%)	0.23
**<60**	17(45.95%)	12(32.43%)	
**Gender**			
**Male**	25(67.57%)	27(72.97%)	0.61
**Female**	12(32.43%)	10(27.03%)	
**Viral infection**			
**Positive**	4(10.81%)	15(40.54%)	0.00
**Negative**	33(89.19%)	22(59.46%)	
**Alcohol consumption**			
**Yes**	12(32.43%)	11(29.73%)	0.80
**No**	25(67.57%)	26(70.27%)	
**Family history**			
**Yes**	7(18.92%)	6(16.22%)	0.76
**No**	30(81.08%)	31(83.78%)	
**Pathology grade**			
**G1/G2**	25(67.57%)	23(62.16%)	0.63
**G3/G4**	12(32.43%)	14(37.84%)	
**TNM Stage**			
**Stage I and II**	12(32.43%)	10(27.03%)	0.61
**Stage III and IV**	25(67.57%)	27(72.97%)	
**AJCC-T**			
**T1-T2**	13(35.14%)	10(27.03%)	0.45
**T3-T4**	24(64.86%)	27(72.97%)	
**TMB level**	3.12(1.24)	6.88(1.82)	0.00

Abbreviation: TMB, tumor mutation burden; AJCC, American Joint Committee on Cancer.

**Figure 1:**
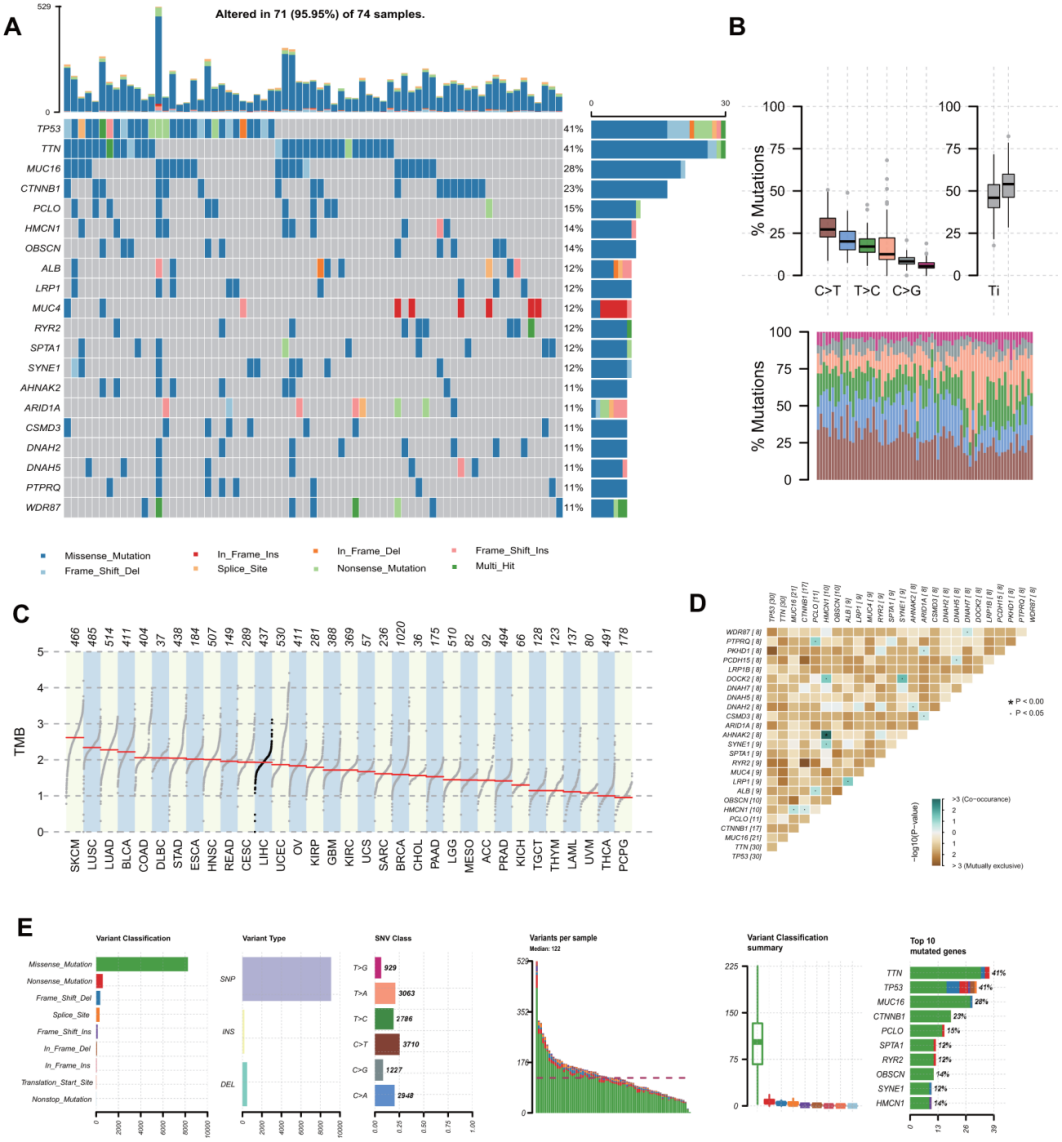
**Landscape of mutation profiles in involved hepatocellular carcinoma tissue samples.** (**A**) Mutation information of top 20 genes in each sample. Different colors and notes at the bottom represented types of mutations. (**B**) Overall distribution of the six different base substitution mutation frequency (above) and conversion ratio in each sample (below). (**C**) Tumor mutational burden ranking of HCC among all tumors in the Cancer Genome Atlas. (**D**) Co-occurrence associations across top 25 mutated genes. (**E**) Summary of the mutation information in all HCC tissue. TMB, tumor mutational burden; SNP, single nucleotide polymorphism; SNV, single nucleotide variants. HCC, hepatocellular carcinoma.

Somatic mutation profiles with VCF format were visualized after propensity score matching (PSM) and X-tile analysis (Figure 1A, Supplementary Figure 1). Genetic information for the Top 20 cumulative mutations were *TP53*, *TTN*, *MUC16*, *CTNNB1*, *PCLO*, *HMCN1*, *OBSCN*, *ALB*, *LRP1*, *MUC4*, *RYR2*, *SPTA1*, *SYNE1*, *AHNAK2*, *ARID1A*, *CSMD3*, *DNAH2*, *DNAH5*, *PTPRQ* and *WDR87*. C>T was the most common single nucleotide mutation (SNV) in HCC (Figure 1B). Base changes in each sample and top 10 mutated genes in HCC were summarized in Figure 1E, and the frequency of single nucleotide polymorphism (SNP) was higher than insertion (INS) or deletion (DEL). Consistent and exclusive association among the mutant genes were shown in Figure 1D, where green represented the co-occurrence relationship and brown represented the exclusion relationship. Figure 1C showed the ranking TMB of HCC among all tumors included in TCGA, which was above average.

**Figure 2:**
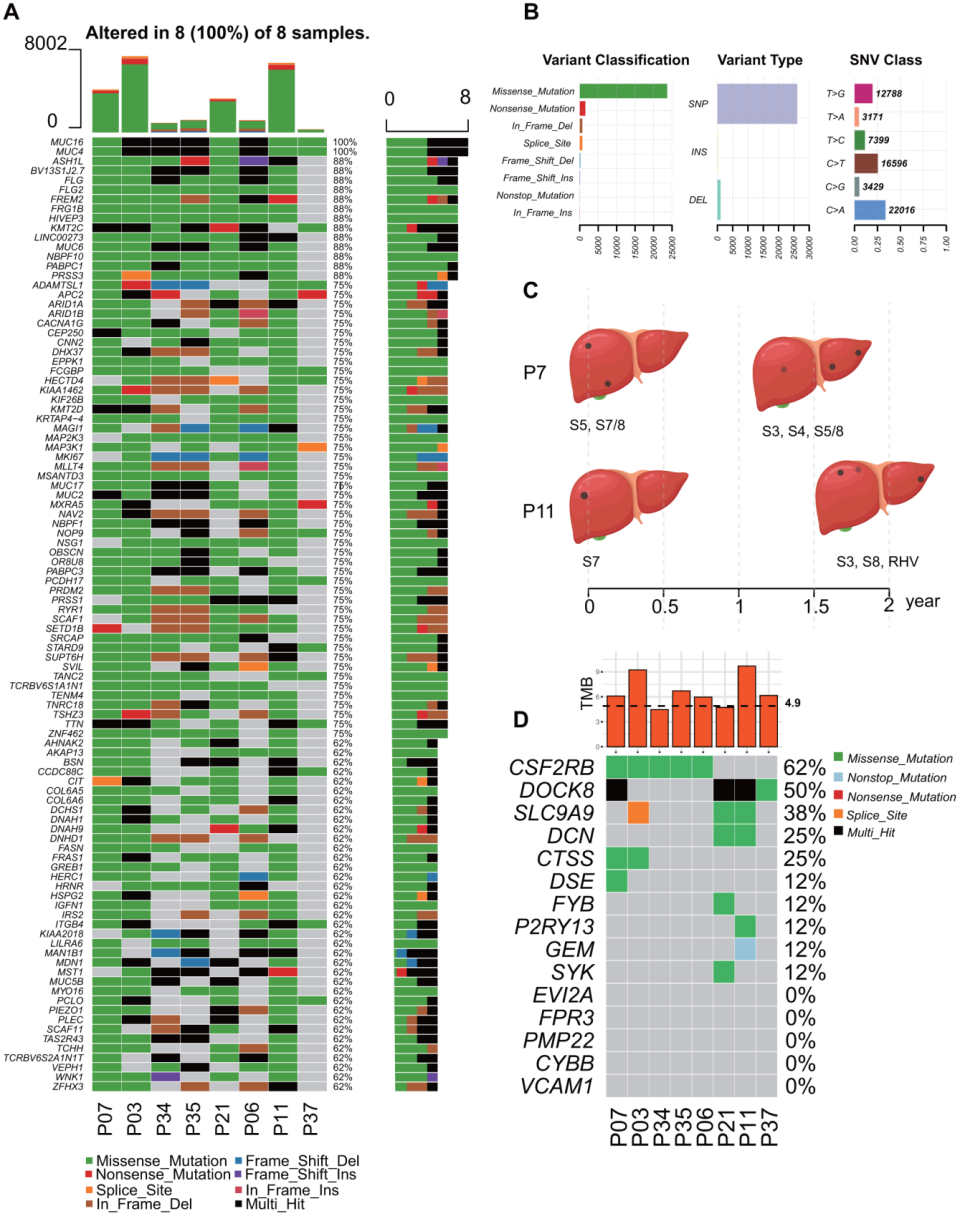
**Landscape of mutation profiles of 8 followed-up HCC patients with recurrence risk.** (**A**) Mutation information of Top 100 genes in each sample. Different colors and notes at the bottom represented types of mutations. (**B**) Summary of variant classification, variant type and SNV class information. (**C**) Time points of the recurrence of P7 and P11, and the location of the lesions. (**D**) TMB distribution of each patients and somatic mutation in immune-related genes among these HCC patients. TMB, tumor mutational burden; IF, Infiltration.

All 8 patients followed up were male patients with chronic hepatitis B (CHB), and Barcelona Clinic Liver Cancer (BCLC) grades were in A~B. Child-Pugh score ranged from 5 to 7, which indicated these patients who received radiofrequency ablations were in good condition. P7 and P11 suffered from recurrence during follow-up, and the lesions were more numerous than before. Clinicopathologic characteristics of the patients were summarized in Supplementary Table 1. Whole-exome sequencing (WES) was applied to the HCC tissues and adjacent normal tissues. Genetic information for the Top 100 cumulative mutations in each sample were showed in Figure 2A. The frequency of SNP was higher than insertion or deletion, and C>A was the more common in these patients (Figure 2B). Time points of P7 and P11 recurrence were shown in Figure 2C, and they were accompanied with higher TMB and more mutations in TMB infiltration (TMB-IF) model (Figure 2D).

### Differential abundance of infiltrating immune cells in low- and high- TMB group

**Figure 3:**
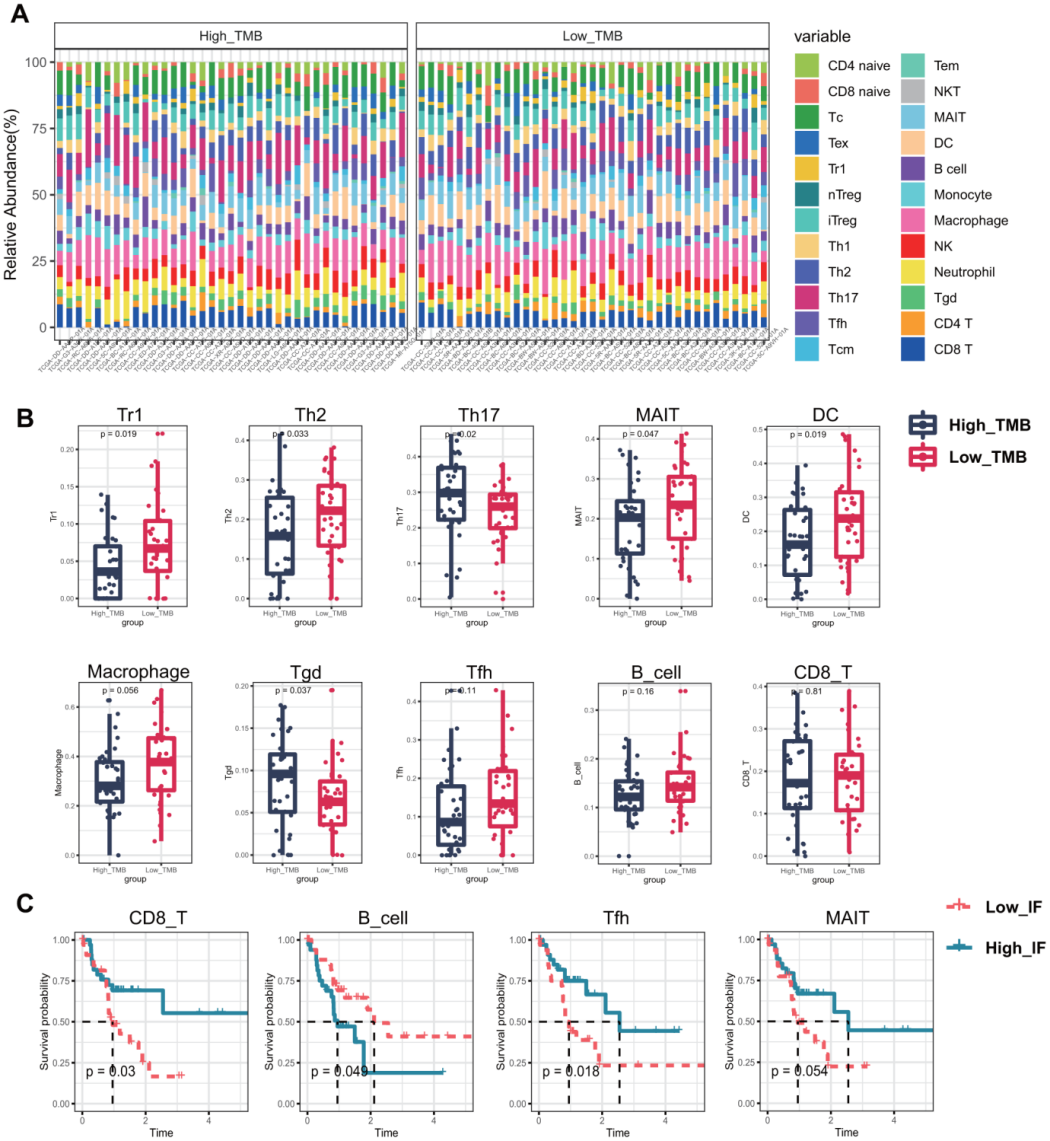
**Infiltrating immune cells between different TMB groups and the effect on overall survival.** (**A**) 24 types of infiltrating cells estimated by ImmuneCellAI. Different colors represent different types of immune cells, and the column length has been normalized by percentage. (**B**) 6 kinds of immune cells with significant infiltration in different TMB groups, and 3 others may affect the survival and prognosis of patients. (**C**) Kaplan–Meier survival analysis identified 4 types of immune cells that may affect the survival status. MAIT, Mucosal associated invariant T cells; Tc, cytotoxic T cells; Tex, exhausted T cells; Tr1, type 1 regulatory cells; nTreg, Natural regulatory T cells; iTreg, Induced regulatory T cells; Th1, Type 1 T helper cells; Th2, Type 2 T helper cells; Th17, Type 17 T helper cells; Tfh, Follicular helper T cells; Tcm, Central memory T cells; Tem, Effector memory T cells; NKT, Natural killer T cells; MAIT, Mucosal-associated invariant T cells; DC, Dendritic cells; Tgd, Gamma-delta T cells; TMB, tumor mutational burden; Low_ IF, low Infiltrating; High_ IF, high Infiltrating.

ImmuneCellAI was adopted to estimate the immune infiltration between low- and high- TMB group. Percentage normalized stack graph showed that immune infiltration scores of the two groups were similar (Figure 3A). Top 10 immune infiltration scores in low TMB group were Macrophage (58.72%), DC (38.57%), Th17 (38.09%), MAIT (36.80%), Tc (34.45%), Th2 (33.77%), iTreg (30.64%), CD8_T cell (29.25%) and NK (25.87%), respectively. Meanwhile, in the high TMB group, the top 10 immune cells were Macrophage (54.40%), MAIT (32.83%), Th17 (51.48%), Tc (37.17%), Th2 (27.98%), iTreg (31.17%), DC (30.24%), NK (29.48%), Neutrophil (32.51%), and CD8_T cell (32.49%), respectively. Percentage table of all immune scores could be found in Supplementary Table 2.

Figure 3B showed the immune cells with different infiltration in the two groups, including Tr1 (P = 0.02), Th2 (P = 0.03), Th17 (P = 0.02), MAIT (P = 0.05), DC (P = 0.02) and Tgd (P = 0.04). Th2, Th17 and Tgd showed higher immune infiltration scores in the high TMB group, while Tr1, MAIT and DC were higher in the low TMB group. The differences of the remaining 14 types of immune cells were all displayed in Supplementary Figure 2A, but they were not significant. To further screen out immune cells with potential impact on the survival of patients, Kaplan-Meier survival curves of all immune cells were plotted based on the median infiltration fraction. And the final potential immune cells were: CD8_T cell (P=0.03), B cell (P= 0.05) and Tfh (P= 0.02) (Figure 3C). Kaplan-Meier survival curves of the remaining 20 meaningless immune cells were shown in Supplementary Figure 2B.

### Comparison of gene expression profiles and functional pathways analysis between low- and high- TMB groups

**Figure 4:**
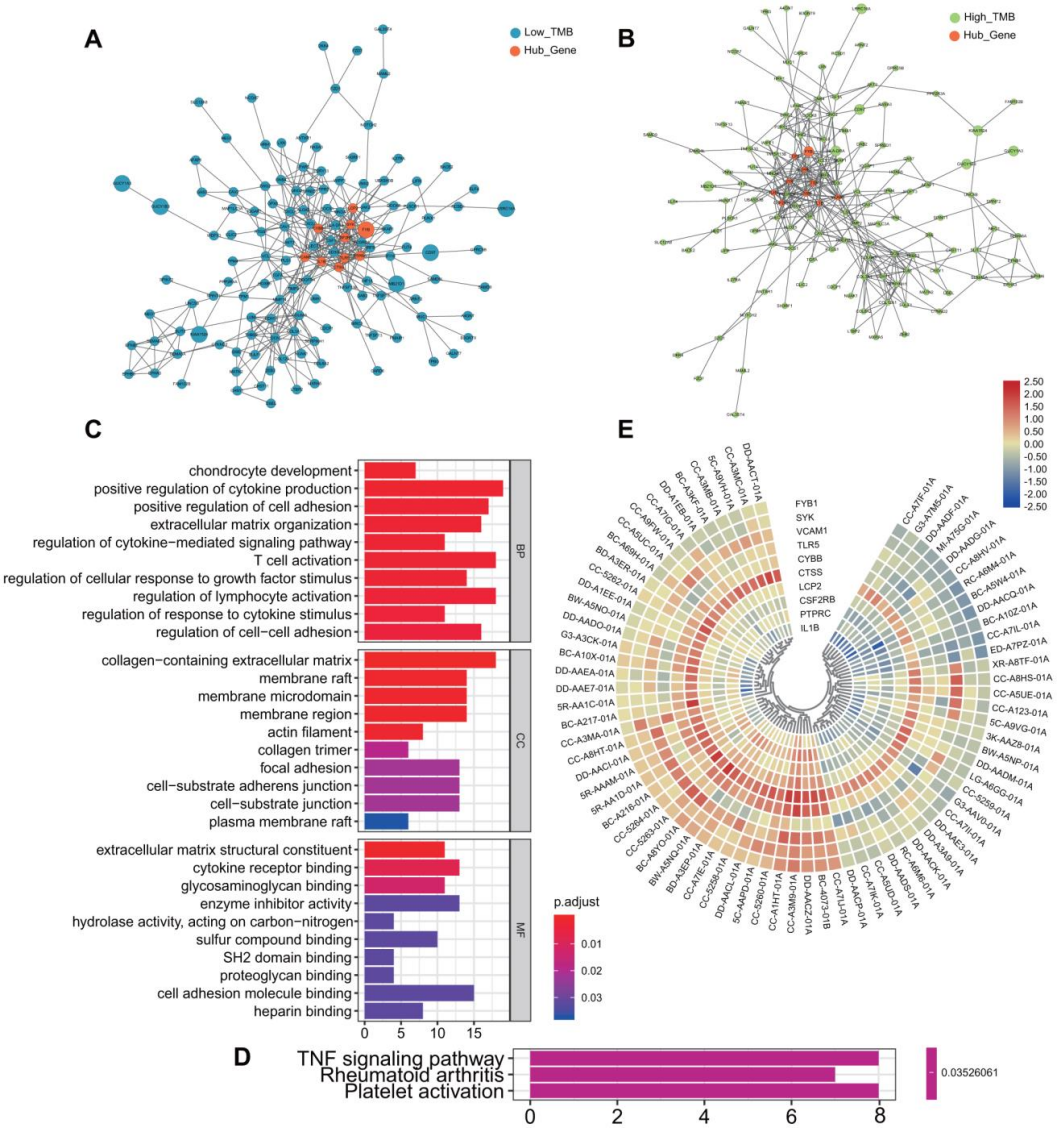
**Differentially expressed genes and functional enrichment analysis between the two groups.** (**A**, **B**) Protein-protein interaction network of all Differentially expressed genes. Orange represents the screened hub genes, and the size of circle represents the level of gene expression. (**C**) Go enrichment analysis of all differentially expressed genes. (**D**) KEGG analysis of all differentially expressed genes: TNF signaling pathway, Rheumatoid arthritis and Platelet activation. (**E**) Heatmap of selected hub genes in each patient. From left to right in order of low TMB and high TMB group. MF, Molecular Function; BP, Biological Process; CC, Cellular Component; GO, Gene Ontology; KEGG, Kyoto Encyclopedia of Genes and Genomes.

All the differentially expressed genes (DEGs) were listed in Supplementary Table 3, from which it can be seen that the expression of most genes was down-regulated with the increase of TMB. Subsequently, we conducted GO enrichment analysis for all of them (Figure 4C). In the part of Biological Process (BP), there were obvious enrichment in the terms of T cell activation and regulation of lymphocyte activation. Other items with potential critical roles included regulation of cell-cell differentiation, positive regulation of cell differentiation, and regulation of response to cytokine stimulus. In the cellular component (CC) part, DEGs were mostly enriched in terms related to cell membrane, such as membrane raft, plasma membrane raft, membrane microdomain, and cell substrata adhesives junction. In Molecular Function (MF), the most enriched terms were the cell adhesion molecule binding, followed by the extracellular matrix structural constituent, cytokine receptor binding and the glycosaminoglycan binding.

KEGG pathway analysis showed that DEGs play an important role in the TNF signaling pathway, which was often required for activated monocytes or macrophage cells to kill or inhibit tumor cells (Figure 4D). Meanwhile, PPI network of DEGs was constructed, and the 10 hub genes were screened out: *PTPRC*, *CYBB*, *CTSS*, *LCP2*, *FYB*, *VCAM1*, *SYK*, *CSF2RB*, *TLR5* and *IL1B* (Figure 4A, 4B). Expression levels of hub genes in each patient were shown by heatmap (Figure 4E). The red part of the figure represents high expression, composed of patients with low TMB.

### Identification of immune-related genes modules and associations of overall survival

**Figure 5:**
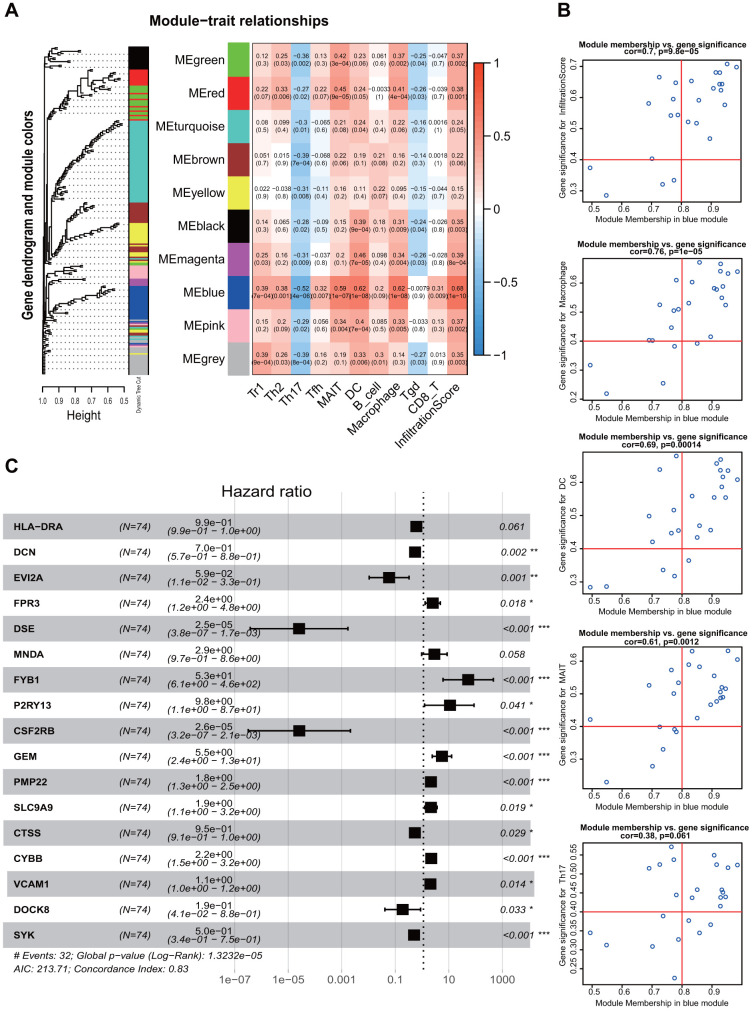
**Weighted Gene Co-expression Network Analysis and screening of immune infiltration and prognostic genes.** (**A**) WGCNA modules related to immune infiltrating cells. Different color represented different modules and correlation was displayed by heatmap. (**B**) Screening of genes associated with immunity and prognosis in the blue module. (**C**) Forest plot shows multivariate regression results and genes that potentially incorporated into the TMB-IF panel. WGCNA, weighted gene co-expression network analysis; TMB-IF, TMB infiltration. Tr1, type 1 regulatory cells; Th2, Type 2 T helper cells; Th17, Type 17 T helper cells; Tfh, Follicular helper T cells; MAIT, Mucosal-associated invariant T cells; DC, Dendritic cells; Tgd, Gamma-delta T cells.

All the immunocytes with different infiltration and those with potential influence on survival were included in weighted gene co-expression network analysis (WGCNA) analysis as immune traits. Then, the correlation matrix of 10 modules of different colors and 10 immune traits with potential influence on prognosis was established (Figure 5A). Macrophage, DC, MAIT and Infiltration Score were well correlated with the genes in the blue module (r=0.62, 0.62, 0.59, 0.68; P < 0.05). Genes screened from the blue module were shown in Figure 5B, and the remaining modules such as red, green, pink and magenta were all exhibited in Supplementary Figure 3. Names of the gene symbol screened from all the modules and their correlation P values were listed in Supplementary Table 4. The best soft Power selected in this study was 13, and relevant pictures was showed in Supplementary Figure 4.

After selection of the genes with good correlation in WCGNA, multivariate Cox regression were performed to screen variables using both the forward and backward likelihood ratio method. 15 genes with statistical significance were screened out: *DCN*, *EVI2A*, *FPR3*, *DSE*, *FYB1*, *P2RY13*, *CSF2RB*, *GEM*, *PMP22*, *SLC9A9*, *CTSS*, *CYBB*, *VCAM1*, *DOCK8* and *SYK* (all P < 0.05) (Figure 5C), which constituted the final TMB-IF panel model.

### Construction and assessment of TMB-IF for HCC

**Figure 6:**
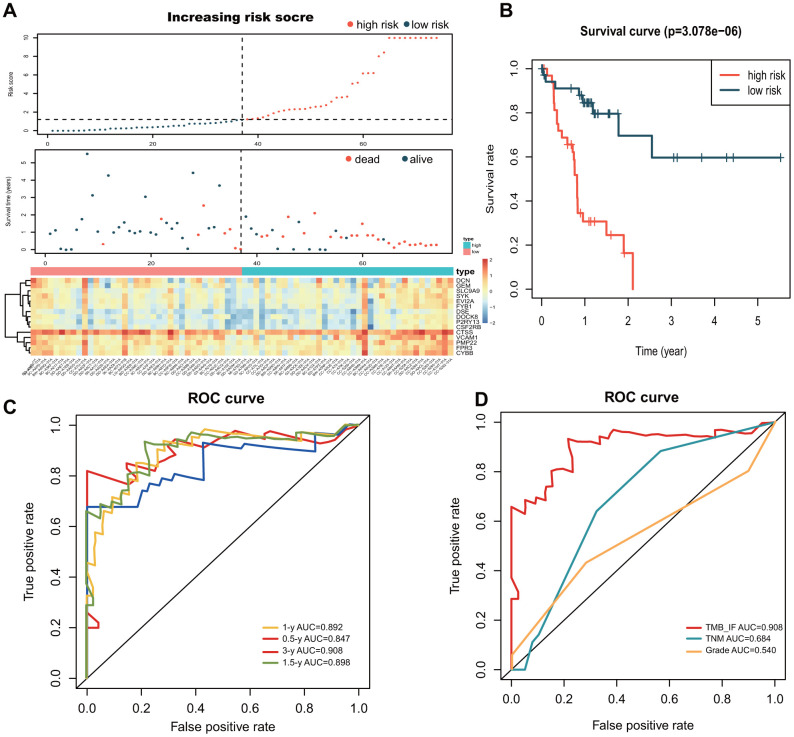
**Construction and evaluation of the TMB-IF prognostic panel.** (**A**) Overall distribution of patients identified with the threshold of high and low risk. Screened prognostic genes in the two groups was demonstrated by heatmap. (**B**) Kaplan–Meier survival analysis and survival curves of high and low risk patients. (**C**) ROC curve of the prognostic model at 0.5-, 1-, 1.5- and 3-year overall survival. (**D**) Comparison of TMB-IF with TNM staging and Pathology Grade. ROC, Receiver operating characteristic; TMB, tumor mutational burden; IF, infiltration.

Patients were segmented to the high- and low- risk groups according to the increased risk score (Figure 6A). Kaplan-Meier survival analysis was re-conducted with the novel panel, and the results showed a good differentiation for patients (P<0.01) (Figure 6B). The formula for calculation was as follows: *h(t)= ho(t) exp (-6.7677CSF2RB - 0.0342CTSS + 0.6675CYBB - 0.2761DCN - 0.7192DOCK8 - 7.4889DSE - 2.2454 EVI2A + 0.8198FPR3 + 1.6468FYB1 + 1.2762GEM + 2.2861P2RY13+0.4223PMP22 + 0.2963SLC9A9 - 0.5453SYK + 0.0848VCAM1).* To determine the predictive accuracy of the TMB-IF, receiver operating characteristic (ROC) curves was drawn (Figure 6C), which demonstrated that the area under the curve (AUC) was 0.85 for 0.5-year survival, 0.89 for 1-year survival, 0.90 for 1.5-year survival, 0.91 for 3-year survival. In addition, we compared the superiority of the novel TMB-IF with the traditional TNM staging with the threshold of 1.5 years. The results showed that the AUC value of TNM was 0.68, which was much lower than that of TMB-IF (0.90 vs 0.68, Figure 6D).

**Figure 7:**
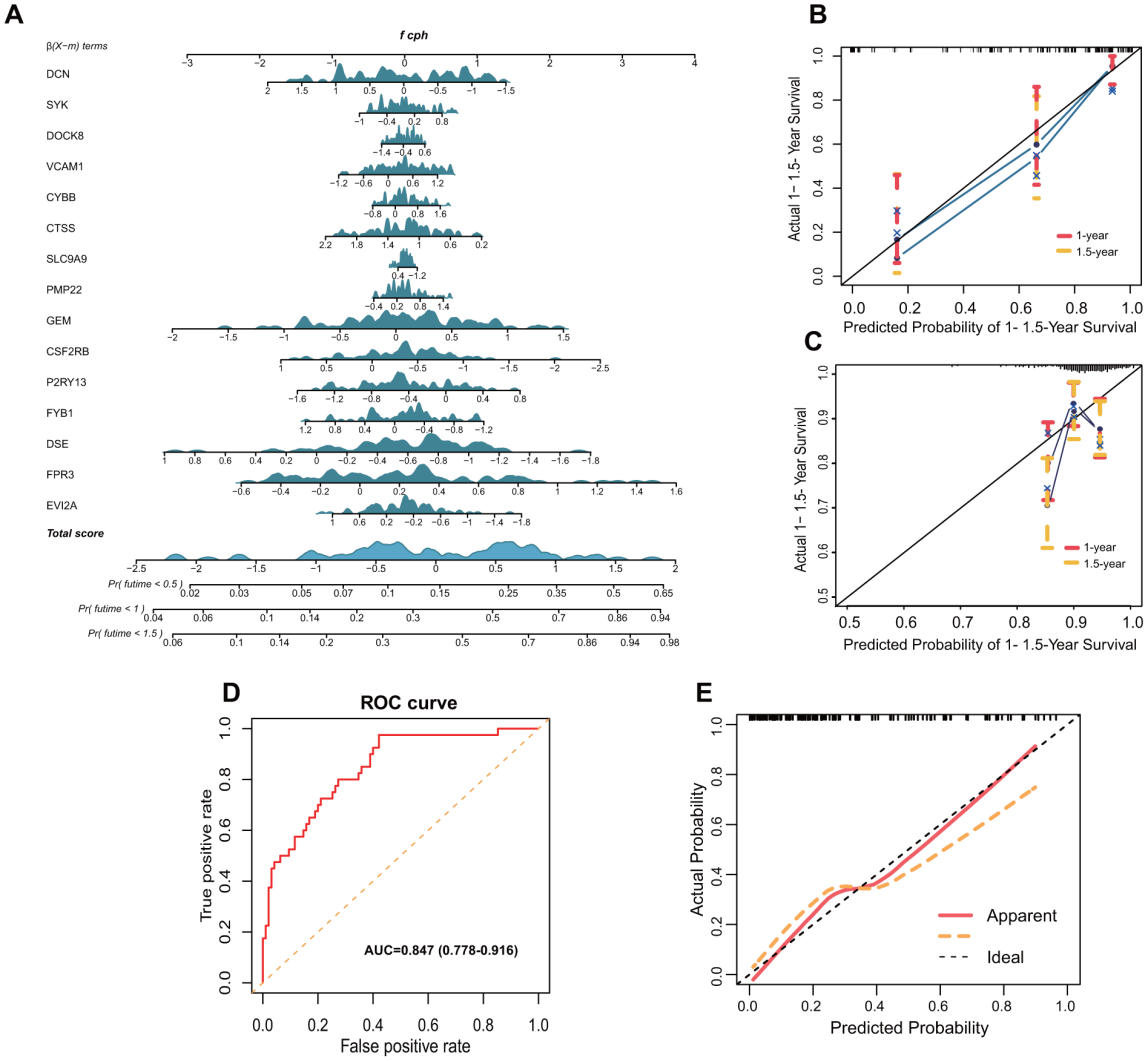
**Nomogram and validation of TMB-IF prognostic panel.** (**A**) Nomogram of all genes that presented significance for prognosis. (**B**) Internal calibration curve of the TMB-IF panel. Red points correspond to the fitting degree of 1-year survival probability, and the orange points correspond to the fitting degree of 1.5-year survival probability. (**C**) Calibration curve of 203 patients in ICGC database. (**D**) ROC curve was utilized to evaluate the potential flexibility of TMB-IF in GSE20017. ROC, Receiver operating characteristic. (**E**) Calibration curve of TMB-IF in predicting tumor vascular invasion.

Prognostic nomogram was shown in Figure 7A. Calibration curve for the probability of survival at 1- and 1.5-year showed optimal consistency between the prediction by TMB-IF and actual observations (Figure 7B), with a C-index of 0.785 (95% CI, 0.700–0.870). The calibration curve of 231 patients from ICGC database was shown in Figure 7C, with a C-index of 0.650 (0.553-0.747). To further explore the extensibility of these screened immune genes, we also tried to apply it to the GSE20017 dataset to investigate the vascular invasion in HCC. The ROC curve of GSE20017 showed the AUC value was 0.847 (95%CI, 0.778-0.916) (Figure 7D). The calibration curve was shown in Figure 7E.

## DISCUSSION

Currently, single-agent PD-1 inhibitors were mainly approved for the second-line treatment of advanced HCC patients with poor response to Sorafenib, which based on two previously reliable clinical studies [5, 24]. Because of the different treatment responses, researchers have been looking for the best biomarkers that can well predict the efficacy of immunotherapy. PD-1/PD-L1 expression level [25], tumor mutation burden (TMB) [13, 26], high microsatellite instability (MSI-H) [27], tumor infiltrating T cell content [28], and neutrophil-lymphocyte ratio (NLR) [29] are currently more widely accepted indicators of response efficacy. Although the predictive value was questioned as well as PD-1/PD-L1 expression level [30], TMB has been proven indeed valuable in predicting the efficacy of immunotherapy by subsequent studies and clinical trials [31].

Although there are few clinical data explaining the role of TMB in HCC, its value can be inferred from other cancer studies. In the clinical study code named CheckMate227, Nivolumab plus low-dose Ipilimumab significantly prolonged the 1-year progression free survival period in advanced non-small cell lung cancer (NSCLC) patients who had TMB≥ 10 mut/MB, regardless of the PD-1 expression level [32]. Meanwhile, CheckMate-568, which was carried out in parallel, verified the conclusion [33]. However, the TMB ≥ 10/MB threshold may not be suitable for HCC. NSCLC ranked second in TMB among all tumors in TCGA database, while HCC ranked 12th. According to the X-tile analysis in this study, the optimal cutoff value of TMB for the two groups of patients was 4.9, which was about half of the threshold of NSCLC. In fact, researchers are trying to circumvent TMB to find ways to enhance the production and expression of neoantigens in tumors, so that patients with lower TMB also can benefit from the immunotherapy [34]. Suggestions proposed now are using the existing chemotherapeutic/targeted agents, radiotherapy, oncological viruses and disrupting endogenous pathways in tumor cells. There is no doubt that these approaches still need further verification.

According to the results of GO enrichment and KEGG analysis of the DEGs in our study, it was noteworthy that the matrix and cell adhesion were involved, including the term of T cell activation that we are concerned with. Recently, osteopontin has been found to regulate the sensitivity of immune cells to PD-1 inhibitors, which was a protein widely distributed in the matrix and cells with function of cell adhesion, signal transduction and tumor metastasis [35]. The colony stimulating factor-1 signaling pathway induced by osteopontin can destroy the transport of tumor-related macrophages and make HCC sensitive to anti-PD-L1 blockade. This suggests that when we pay more attention to the immune cells, perhaps matrix components in the background environment are subtly influencing the immunocyte migration. As for top 10 selected hub genes finally fit into TMB-IF, key genes were identified as *FYB*, *CSF2RB*, *CTSS*, *CYBB*, *VCAM1* and *SYK*. Cathepsin S silencing (*CTSS*) has been proved to have a potential role of inducing apoptosis of HCC cells [36]. *VCAM1*, a gene associated with vascular cellular cytokines molecule, has been suggested that it may endue the growth and infiltration capacity of HCC cells [37]. And spleen tyrosine kinase (*SYK*), which plays an important role in immune cell signaling pathway, has been reported as a biomarker for HCC and a potential target role for liver fibrosis [38]. However, there is no research on *FYB*, *CSF2RB* and *CYBB* genes in HCC at present. Other component genes of the TMB-IF panel included *DCN*, *DSE*, *EVI2A*, *FPR3*, *P2RY13*, *GEM*, *PMP22*, *SLC9A9* and *DOCK8*. *DCN* has been reported to be associated with DNA methylation and hydroxy-methylation in HBV-related HCC [39], and *DSE* dysregulation may contributed to the malignant behavior of HCC cells through suppression of *CCL5* signaling [40]. However, the remaining genes are rarely reported in HCC and still require further basic research.

One of the characteristics of HCC is the rich blood supply and early blood-borne metastasis [41]. To further improve the response of PD-1 inhibitors, it is necessary to understand the mechanism of abnormal PD-1/PD-L1 signaling and immune escape microenvironment. Tumor infiltrating lymphocytes (TILs) are representative components of host anti-tumor immune response [42]. CD3, CD4, CD8 and Foxp3 positive T lymphocytes are the most common subsets of TILs. Among them, tumor infiltration of CD8 positive T cells plays the most important role in host immune defense against tumor progression. In fact, patients in the high TMB group of our study did not showed the up-regulation in cytotoxic T lymphocytes as we expected. Our study demonstrated the infiltration abundance of Th2, Th17 and Tgd increased in the high TMB group, while Tr1, MAIT and DC increased in the low TMB group. Survival analysis showed that patients in the Low-TMB group had better survival outcomes, which was speculated might be related to increased infiltration of DC and MAIT cells through previous studies. More DC cells mean a greater chance of exposure of killer T cells to neoantigens. Another immune cell enriched in the low TMB group were Tr1 cells, which induce and maintain immune tolerance and immune regulatory cells. However, studies have shown that plasmacytoid dendritic cells (pDCs) promote the role of Tr1 cells in tumor immunosuppression in HCC [43], which may interfere with the efficacy of immunotherapy. MAIT cells are naturally equipped with anti-tumor function, but if the function of HCC infiltrated MAIT cells are impaired or recoded, the direction of anti-tumor immunity will be shifted to tumor promotion [44]. In the high TMB group, increased Th2 broke the balance of Th1/Th2, which is one of the poor prognostic factors in HCC patients [45]. And Th17 is a subset of inflammatory T helper cells that has been showed to potentially stimulate the development of HCC [46]. Gamma-delta T cells (Tgd) possess unique characteristics and antigen recognition ability, as well as unique tissue affinity and cytotoxicity, which enable these cells to induce long-lasting immunity in response to different pathological conditions [47]. Therefore, Tgd cells are more likely to play an important role when the immunosuppressive state of HCC broken by PD-1 inhibitors.

A recent study by Shinji Itoh et al. confirmed the presence of vessels that encapsulated the tumor cluster (VETC) was associated with a high positive rate of PD-1 in HCC tissues, successfully established a link of PD-1, tumor vascular and immunotherapy [48]. Therefore, combination of PD-1 inhibitors and anti-angiogenic drugs are expected to significantly improve the response rate of immunotherapy in HCC patients [49]. It is for this reason that we have tried to apply TMB-IF to predict vascular tumor invasion, and the good discrimination was showed. Then, encouraged by the above performance, we further explore the potential application of TMB-IF in the recurrence of early stage of HCC. Although limited by the small sample size, it also can be seen that relapsed patients have higher levels of TMB than those without recurrence, and indeed have more mutations in immune-related genes. Previous studies have confirmed a high rate of recurrence after radiofrequency ablation, with nearly half of people relapsed within 3 years [50]. And recent researches suggested that it was related to a late dynamic of immature NK cells (CD56+) and altered myeloid DC (PD-L1+) [51]. Therefore, it is possible that PD-1/PD-L1 inhibitors will also play a role in preventing early recurrence of HCC. In the coming era of "immunotherapy plus", PD-1 inhibitors may have a broader indication than current clinical studies of advanced HCC, and more researches are needed in the future.

There are some limitations exist in this study: (I) basic experiments are needed to verify the correlation between gene signature and immune cell infiltration; (II) more clinical samples are needed to verify the prognostic effect of TMB-IF and its potential relationship with immune infiltration, vascular invasion and tumor recurrence.

In conclusion, higher TMB was associated with worse survival outcomes. The TMB-IF panel based on tumor mutation burden and immune cell infiltration has demonstrated the stability and scalability of discrimination. Additionally, PD-1 inhibitors may have a wider application in HCC in the future, hoping to reduce the high recurrence rate after radical treatment. The intricate relationship between immune cells, tumor cells and immunotherapy still need to be further investigated.

## MATERIALS AND METHODS

### Somatic mutation, transcriptome, microarray data acquisition and pre-processing

Somatic mutation data of 374 LIHC patients were downloaded from the publicly available TCGA database through the GDC data portal (https://portal.gdc.cancer.gov/), corresponding data format was based on the VarScan software platform “Masked Somatic Mutation”. MiRNA expression profiling, clinical data including age, gender, tumor grade, pathological stage, AJCC-TNM stages and survival outcomes were all download for uniqueness matching. Meanwhile, miRNA expression files and related clinical outcome data of 203 HCC patients from Japan were also downloaded from ICGC official website (https://dcc.icgc.org/). In addition, gene expression chip profiles of GSE20017 (n=135) were also derived from the Gene Expression Omnibus (GEO; http://www.ncbi.nlm.nih.gov/geo/). Along with the microarray data, corresponding platforms files "GPL8432" were also downloaded.

Mutation Annotation Format (MAF) of somatic variants downloaded from TCGA were visualized using "maftools" R package. Uniqueness matching with transcriptome data, collating ICGC gene expression profiling and GEO microarray data were all done using the Perl script (Perl version 5.28). TMB was defined as the total number of somatic gene coding errors detected per million bases, including base substitution, insertion, or deletion. Mutation frequency with number of variants/the length of exons (38 million) of 374 HCC patients were all calculated. Patients with well-matched clinical outcomes were subjected to Kaplan-Meier analysis followed by X-tile analysis [52] to determine the best TMB cutoff. Then, according to age, gender and pathology grade, the two groups of high and low TMB were matched by the propensity score matching (PSM) in SPSS software. Finally, the training data set of the panel was selected.

### Patients followed-up and samples involved

Eight patients diagnosed with HCC who underwent radiofrequency ablation during November 2017 and August 2018 in the third Affiliated Hospital of Sun Yat-sen University were enrolled. The operation was performed by two attending physicians with more than five years' experience. A total of 8 paired samples of liquid nitrogen quick-frozen HCC tissue that had been histologically and clinically diagnosed were harvested in this current study. Normal liver tissue from 2cm past the tumor edge were collected for controls. Each sample was immediately immersed in liquid nitrogen after isolation and then transferred to -80° C within 30 minutes for later whole-exome sequencing (WES). Prior patients’ consent and approval of the Institutional Research Ethics Committee of the Third Affiliated Hospital were obtained for the regular follow-up. Postoperative tumor status and recurrence will be recorded.

### Differentially expressed genes and functional enrichment analysis

According to the results of X-tile analysis and PSM, transcriptome data of 374 HCC patients were divided into high TMB group and low TMB group via R software. "limma" R package was utilized to identify differentially expressed genes (DEGs) between the two groups. To avoid missing potentially important immune-related differential genes, we set the screening criteria at |Fold Change (FC)| >1 and False Discovery Rate (FDR) <0.05, which was also conducive to further weighted gene co-expression network analysis (WGCNA). After screening out DEGs, we used "org.HS.Eg.db", "clusterProfiler", "enrichplot", "ggplot2" packages to implement the Gene Ontology (GO) enrichment analysis of DEGs. Similarly, Kyoto Encyclopedia of Genes and Genomes (KEGG) pathway analysis of the DEGs was also achieved by the above 4 R packages. Preliminary Protein-Protein interaction (PPI) networks between the differential genes were explored by STRING online database (http://string-db.org). Subsequently, the file containing nodes information was imported into Cytoscape software (version 3.6.1) for PPI network plotting. Top 10 nodes ranked by degree were filtered out using CytoHubba plug-in.

### ImmuneCellAI and prognostic analysis of immune cells

After comprehensive consideration of TIMER database [53] and CIBERSORT algorithm [54], we further conducted immune cell infiltration analysis in ImmuneCellAI [55], which demonstrated powerful and unique function in tumor immune infiltration estimation and immunotherapy response prediction (http://bioinfo.life.hust.edu.cn/ImmuCellAI/). Transcriptome data from both groups were submitted to ImmuneCellAI, and all original matrices containing infiltrating immune cells information were downloaded. All boxplots were drawn with "ggpubr" R package, and bilateral Wilcoxon rank-sum test and P value were used to compare the differences between the two groups. Then, scores of all immune cells were normalized by percentage, a stack graph was drawn using the "ggplot2" R package. Kaplan-Meier analysis for each type of immune cells showed differences in survival outcomes at different levels of infiltration. R package utilized was "survival", and the log-rank test of P< 0.05 was considered statistically significant.

### WGCNA and tumor immune related gene identification

After screening out potential key immune cells, they were included as independent immune traits for weighted gene co-expression network analysis (WGCNA). Background genes were differentially expressed genes, and the R package involved was “WGCNA”. Power parameter was predicted through the pickSoftThreshold function, which can provide appropriate soft threshold power for network construction by calculating scale-free topology fitting index of multiple powers. CutreeDynamic function was used to trim the gene level cluster tree, and then the co-expression module was obtained. ModuleEigengenes function in R WGCNA package was used to calculate the differences between the feature genes of each module. Association between modules and immune traits was assessed by Pearson’s correlation. And we further extracted immune cell-related genes under the conditions of Module Membership > 0.8 and Gene Significance correlation > 0.4. Genes from statistically significant modules were further incorporated into the multivariate Cox regression to screen genes related to prognosis.

### TMB-IF gene panel generation and verification

Both "LR forward" and "LR backward" were used for variable filtering, and all independent prognostic factors determined by multivariate Cox regression analysis were included to establish the TMB-Infiltration (TMB-IF) panel to investigate the probability of 0.5-, 1- and 1.5- year overall survival (OS). Concordance index (C-index) was calculated to assess the consistency between the actual observations and probability predicted, with bootstrap method of 1000 resamples. Receiver operating characteristic (ROC) curves were performed to compared the identification of TMB-IF predictions of 0.5, 0.5-, 1-, 1.5- and 3- year OS, using the “survivalROC” R package. Then, the 1.5-year OS predictions of TMB-IF and TNM stages were compared, ROC curves were drawn, and AUC values were calculated. The remaining two validation cohorts (ICGC-JP, GSE20017) were used similar approach to validate the model. Nomogram and calibration diagrams involved were drawn using the “rms” R package.

### Statistical analysis

Statistical significance of differences among variables with normal distribution was estimated by Student's t-test, while non-normal distribution variables were analyzed by Mann-Whitney U test. Qualitative variables were analyzed by Pearson χ2 test or Fisher’s exact test. Correlation was calculated using Pearson’s and distance correlation analysis. Survival probability was calculated by Kaplan–Meier method, and Log-rank test was used to test the significance of differences in survival curves. Multivariate analysis adopted Cox proportional hazards regression model, and methods of variable filtering were likelihood ratio test of maximum partial likelihood estimation (both forward: LR and backward: LR). Accuracy of survival prediction was evaluated by receiver operating characteristic curve (ROC) analysis and Harrell's concordance index (C-index) analysis. All statistical analyses were performed using R software (version 3.6.2) and SPSS software (version 26.0). Tow-tailed P < 0.05 was considered statistically significant.

### Data accessibility

Sample collection, DNA extraction and Whole exome sequencing (WES) of follow-up patients were available in Supplementary Materials and Methods.

All data generated and analyzed during this study are available from public database (see Materials and Methods). The raw Illumina read data for all samples were deposited in the National Center for Biotechnology Information Sequence Read Archive database under the accession number PRJNA632989.

### Ethics approval and consent to participate

This study complies with the Declaration of Helsinki. Prior patients’ consent and approval of the Institutional Research Ethics Committee of the Third Affiliated Hospital has been obtained for the use of the clinical materials.

## Supplementary Material

Supplemental Materials and Methods

Supplementary Figures

Supplementary Tables 1 and 2

Supplementary Table 3

Supplementary Table 4
