# Supplementary material for: A novel panel based on immune infiltration and tumor mutational burden for prognostic prediction in hepatocellular carcinoma
Source: Aging (Albany NY). 2021 Mar 10;13(6):8563–87. doi: 10.18632/aging.202670 (PMC8034943; doi:10.18632/aging.202670)
Supplement: Supplemental Materials and Methods [file aging-13-202670-s001.pdf]

SUPPLEMENTARY MATERIALS

Supplementary Materials and Methods

Sample collection and DNA extraction

Matched HCC tissue and adjacent normal tissue from 8 patient were harvested by biopsy prior before radiofrequency ablation. DNeasy Blood and Tissue Kit (Qiagen, Germany) was utilized to extract and purify DNA from the tissues according to the manufacturer's instructions. Concentration and quality of the DNA were determined using a NanoDropND-1000 spectrophotometer. 0.8% agarose gel electrophoresis was performed to confirm the quality of the DNA. All samples passed strict quality supervision tests and were available for Whole exome sequencing (WES).

Whole exome library preparation and sequencing

Qualified genomic DNA of tumor and matched adjacent normal tissue were fragmented to 200-300 bp by Covaris technology with resultant library, and then adapters were ligated to both ends of the fragments. Next, extracted DNA was amplified by ligation-mediated PCR, purified and hybridized to the Agilent human exome array for enrichment. Non-hybridized fragments were washed out. All products were subjected to real-time PCR to estimate the magnitude of enrichment. Finally, captured library was loaded on a HiSeq 2500 platform, and sequences of each individual were generated as 150bp paired-end reads. All sequencing processes were controlled by data collection software according to the Illumina HiSeq 2500 Users' Manual.

Exome sequencing mapping and variation detection

Paired-end reads to the NCBI human reference genome (hg19) was aligned using the BWA(1) (Burrows–Wheeler Alignment, version 5.9) software with default parameters. Aligned BAM files of each library were then sorted and merged using Samtools 0.1.19. Flagstat tool was utilized to assess the mapping information. Local realignment of the original BAM alignment was performed using the GATK(2) (Genome Analysis Toolkit v 4.0.8.1), followed by Picard to mark duplicates reads. MuTect2 was used to detect somatic mutation based on BWA alignment. If the following criteria are met, the highly reliable somatic SNVs (single nucleotide variations) will be called: (I) both tumor and normal samples should be covered sufficiently at the genomic position ($\geq 10\times$); (II) the mutation should be supported by at least 5% of the total reads in the tumor, but less than 1% in the normal; (III) the variants should be supported by at least five reads in the tumor. Then, INDELs (insert and deletion mutations) were processed: (I) candidate somatic indels were predicted by GATK Somatic Indel Detector with default parameters; (II) All the somatic mutations were screened out by the dbSNP (version 147) site, which was commonly polymorphic without known medical effect. Finally, the remaining mutations were annotated with ANNOVAR(3) for subsequent analysis.
