# Supplementary material for: A novel panel based on immune infiltration and tumor mutational burden for prognostic prediction in hepatocellular carcinoma
Source: Aging (Albany NY). 2021 Mar 10;13(6):8563–87. doi: 10.18632/aging.202670 (PMC8034943; doi:10.18632/aging.202670)
Supplement: Supplementary Figures [file aging-13-202670-s002.pdf]

SUPPLEMENTARY FIGURES

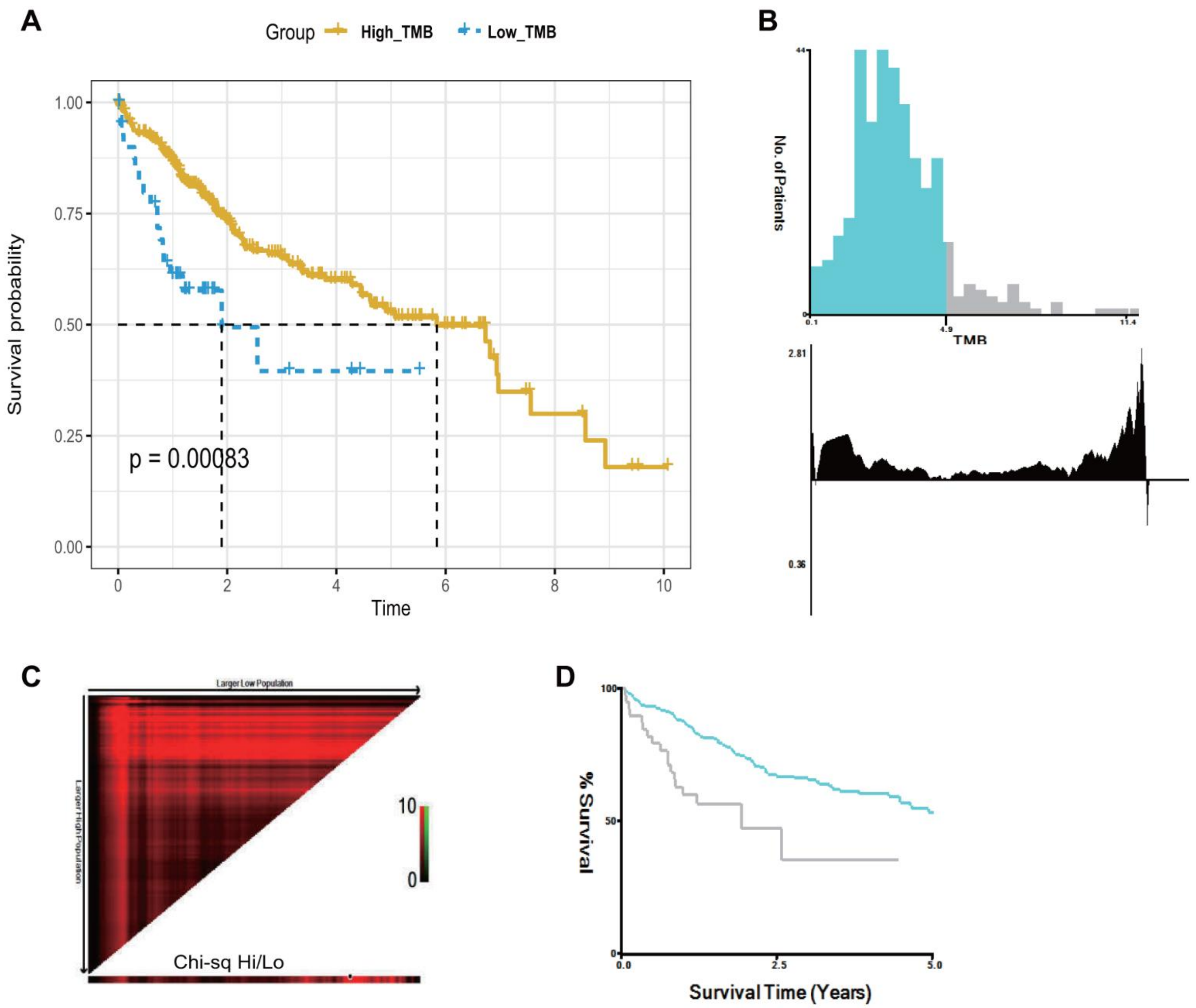

Supplementary Figure 1. Results of X-tile analysis of 374 HCC patients in TCGA database. (**A**, **D**) Survival curve after the optimal cutoff value was calculated by X-tile analysis, P<0.001. (**B**, **C**) Risk distribution, and the determination of best cutoff value of tumor mutation burden (TMB).

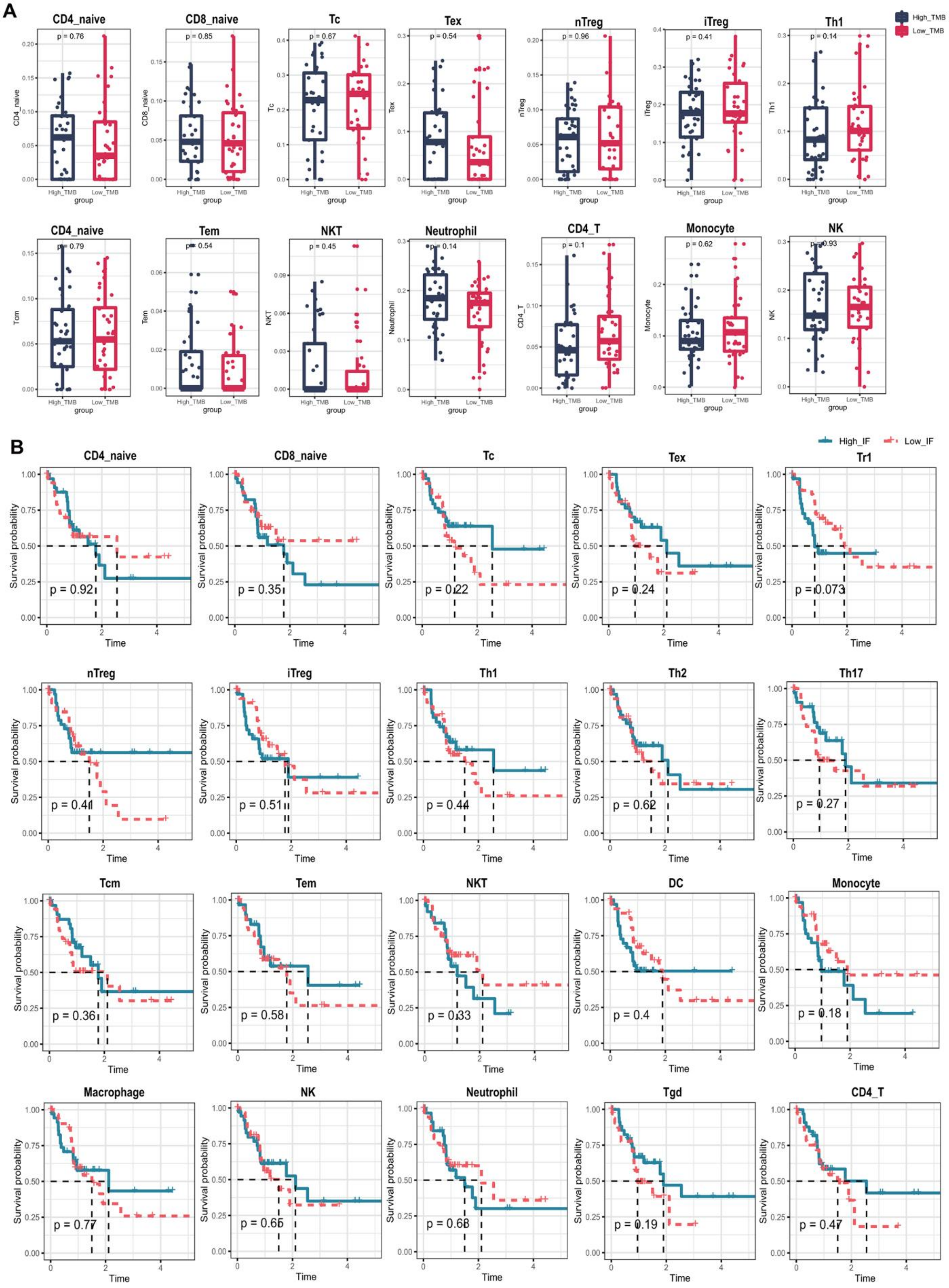

Supplementary Figure 2. The remaining types of immune infiltrating cells between different groups and survival analysis. (**A**) 14 types of immune cell infiltration, in addition to the immune cells listed in Figure 2. (**B**) Survival analysis of other immune infiltrating cells, in addition to the five immune cells shown in Figure 2.

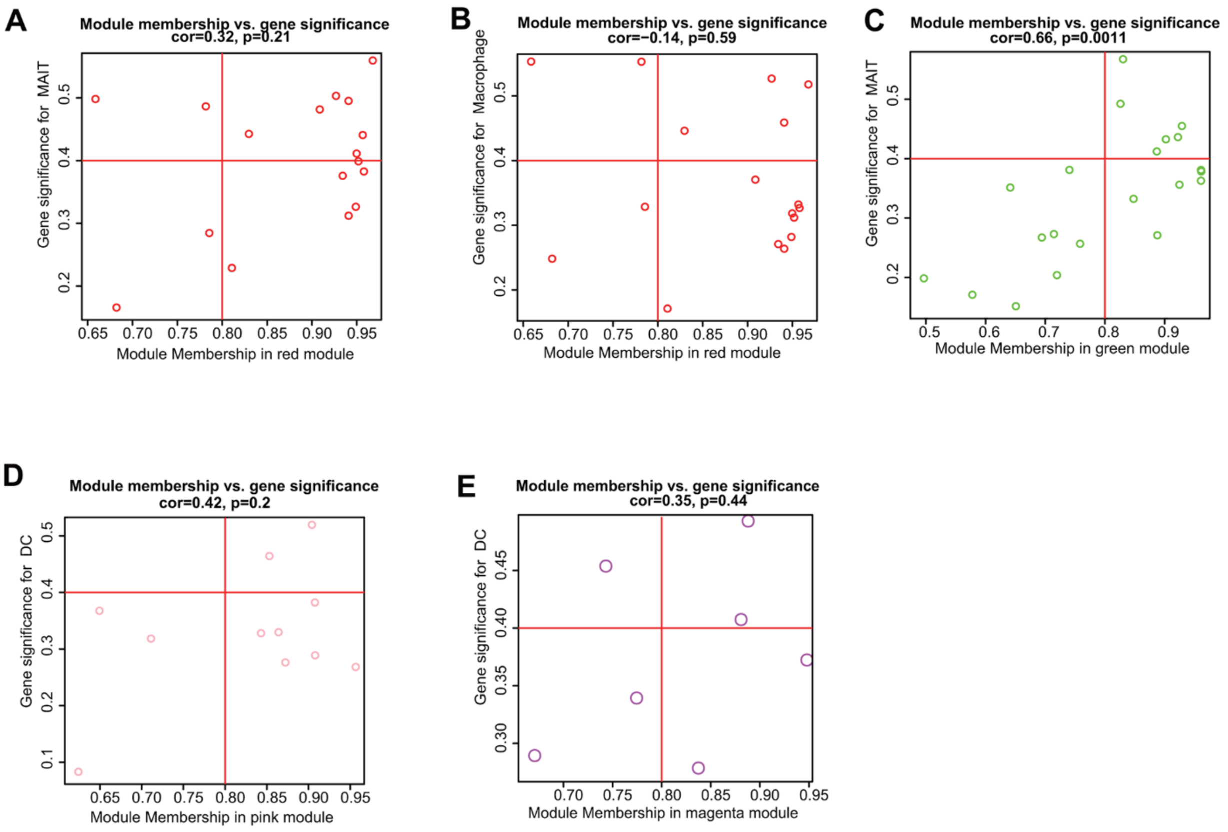

Supplementary Figure 3. Immune related gene modules of WGCNA analysis. (**A**–**E**) Immune related genes in red, green, pink and magenta modules.

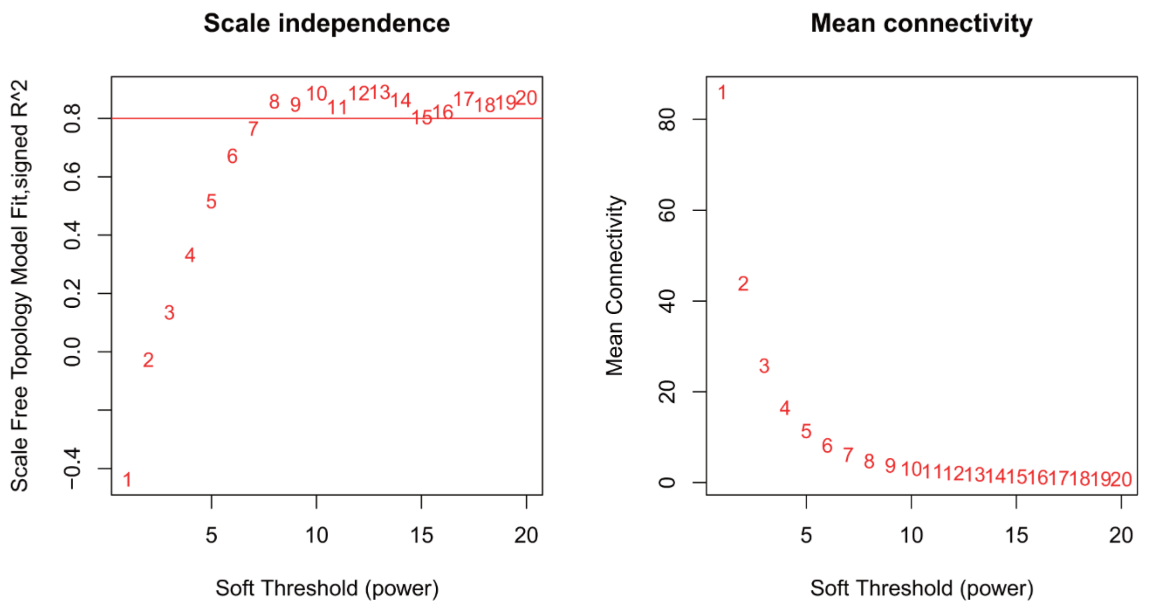

Supplementary Figure 4. The soft threshold selected in this experiment in the process of WGCNA analysis.
