# Supplementary material for: A novel panel based on immune infiltration and tumor mutational burden for prognostic prediction in hepatocellular carcinoma
Source: Aging (Albany NY). 2021 Mar 10;13(6):8563–87. doi: 10.18632/aging.202670 (PMC8034943; doi:10.18632/aging.202670)
Supplement: Supplementary Tables 1 and 2 [file aging-13-202670-s003.pdf]

SUPPLEMENTARY TABLES

Supplementary Table 1. Clinical and demographic information of HCC samples involved in the study.

Parameters	P3	P7	P11	P21	P6	P34	P35	P37
**Gender**	Male	Male	Male	Male	Male	Male	Male	Male
**Operation date**	2017-11-14	2017-11-30	2017-12-12	2018-4-17	2017-11-28	2018-7-12	2018-7-17	2018-8-14
**Age**	58	52	60	62	55	58	38	54
**Smoking**	No	No	No	Yes	Yes	No	Yes	Yes
**Alcohol consumption**	No	No	No	Yes	No	No	Yes	No
**Hypertension**	No	Yes	No	Yes	Yes	No	No	No
**Diabetes**	Yes	Yes	No	No	No	No	No	No
**Family history**	No	No	No	No	No	No	No	No
**Viral hepatitis**	HBV	HBV	HBV	HBV	HBV	HBV	HBV	HBV
**HBV-DNA (IU/ml)**	5.41E+02	1.35E+04	<2.00E+1	7.04E+02	4.92E+05	<100	4.91E+04	2.19E+01
**AFP (ng/ml)**	2.19	10.6	17.11	419.92	186.1	0.8	10.857	1.677
**Cirrhosis**	No	Yes	Yes	No	No	No	Yes	Yes
**Tumor number**	1	2	1	4	2	1	1	1
**BLCL stage**	A	A	A	B	A	A	A	A
**Child-Pugh score**	5	5	7	5	5	5	5	5
**Recurrence**	-	2019-04-10	2020-01-20	-	-	-	-	-

Supplementary Table 2. Scores of immune cells between the two groups.

Immune cells	Low TMB (Mn±SD) (%)	Percentage (%)	High TMB (Mn±SD) (%)	Percentage (%)
CD4_naive	0.05±0.06	8.67%	0.06±0.05	10.29%
CD8_naive	0.05±0.05	8.38%	0.05±0.04	9.54%
Tc	0.22±0.11	34.45%	0.21±0.12	37.17%
Tex	0.07±0.09	11.54%	0.08±0.08	14.49%
Tr1	0.07±0.06	11.55%	0.04±0.04	7.47%
nTreg	0.06±0.05	9.49%	0.06±0.04	10.01%
iTreg	0.19±0.09	30.64%	0.17±0.08	31.17%
Th1	0.11±0.08	18.16%	0.09±0.07	15.94%
Th2	0.21±0.1	33.77%	0.16±0.11	27.98%
Th17	0.24±0.08	38.09%	0.29±0.11	51.48%
Tfh	0.15±0.11	24.00%	0.11±0.1	20.11%
Tcm	0.06±0.05	9.48%	0.06±0.04	10.24%
Tem	0.01±0.01	1.55%	0.01±0.02	2.41%
NKT	0.01±0.03	2.04%	0.02±0.03	3.37%
MAIT	0.23±0.1	36.80%	0.18±0.1	32.83%
DC	0.24±0.13	38.57%	0.17±0.11	30.24%
B_cell	0.15±0.06	23.19%	0.13±0.05	22.57%
Monocyte	0.11±0.06	17.79%	0.1±0.05	18.33%
Macrophage	0.37±0.15	58.72%	0.3±0.13	54.40%
NK	0.16±0.07	25.87%	0.16±0.07	29.48%
Neutrophil	0.16±0.06	24.88%	0.18±0.06	32.51%
Tgd	0.07±0.04	10.48%	0.09±0.05	15.60%
CD4_T	0.07±0.04	10.76%	0.05±0.04	9.00%
CD8_T	0.18±0.09	29.25%	0.18±0.1	32.49%

Abbreviation: MAIT, Mucosal associated invariant T cells; Tc, cytotoxic T cells; Tex, exhausted T cells; Tr1, type 1 regulatory cells; nTreg, Natural regulatory T cells; iTreg, Induced regulatory T cells; Th1, Type 1 T helper cells; Th2, Type 2 T helper cells; Th17, Type 17 T helper cells; Tfh, Follicular helper T cells; Tcm, Central memory T cells; Tem, Effector memory T cells; NKT, Natural killer T cells; MAIT, Mucosal-associated invariant T cells; DC, Dendritic cells; Tgd, Gamma-delta T cells; TMB, tumor mutational burden; Low_ IF, low Infiltrating; High_ IF, high Infiltrating.
