# Supplementary material for: A novel panel based on immune infiltration and tumor mutational burden for prognostic prediction in hepatocellular carcinoma
Source: Aging (Albany NY). 2021 Mar 10;13(6):8563–87. doi: 10.18632/aging.202670 (PMC8034943; doi:10.18632/aging.202670)
Supplement: Supplementary Table 3 [file aging-13-202670-s004.docx]

**Supplementary Table 3. Differential immune genes between low TMB and high TMB groups**

| Gene | Low TMB | High TMB | logFC | pValue | FDR |
| --- | --- | --- | --- | --- | --- |
| IL27RA | 3.722416 | 1.706639 | -1.12508 | 0.001996 | 0.047096 |
| ST8SIA4 | 0.982911 | 0.401716 | -1.29088 | 0.000862 | 0.037896 |
| CARD6 | 2.315503 | 1.012231 | -1.19379 | 0.001349 | 0.041893 |
| HLA-DRA | 381.3331 | 160.8658 | -1.24519 | 0.001996 | 0.047096 |
| GAS7 | 1.541713 | 0.396826 | -1.95795 | 0.001059 | 0.039132 |
| SLC49A3 | 3.386339 | 1.525202 | -1.15073 | 0.000518 | 0.035979 |
| ANTXR1 | 3.55796 | 0.869035 | -2.03356 | 0.000211 | 0.032229 |
| PPP2R3A | 0.869752 | 0.30097 | -1.53099 | 0.001196 | 0.040614 |
| MMP14 | 56.34482 | 10.90443 | -2.36937 | 3.76E-05 | 0.024487 |
| EFNB1 | 11.70013 | 5.613133 | -1.05965 | 0.000565 | 0.036237 |
| IL1B | 0.780854 | 0.227688 | -1.77799 | 0.000496 | 0.035979 |
| TIMP2 | 27.79456 | 12.37986 | -1.16681 | 0.002239 | 0.048713 |
| GNB4 | 1.682254 | 0.701518 | -1.26184 | 0.002239 | 0.048713 |
| DIO3 | 1.001258 | 0.08001 | -3.64549 | 0.00052 | 0.035979 |
| FZD7 | 3.333225 | 0.735096 | -2.18091 | 0.002074 | 0.047802 |
| SLC6A13 | 0.723777 | 2.294055 | 1.664283 | 0.001996 | 0.047096 |
| PIMREG | 1.903073 | 0.588514 | -1.69318 | 0.001103 | 0.039945 |
| VCL | 7.989277 | 3.295194 | -1.2777 | 0.001149 | 0.04026 |
| PRKX | 1.603586 | 0.634721 | -1.33711 | 0.001059 | 0.039132 |
| SWAP70 | 5.623853 | 2.638721 | -1.09172 | 0.000145 | 0.032229 |
| CLIC2 | 3.858489 | 1.798335 | -1.10137 | 0.000254 | 0.032229 |
| TNFSF13 | 1.51981 | 0.720258 | -1.0773 | 0.000699 | 0.036953 |
| DCN | 7.24351 | 1.490207 | -2.28118 | 0.001349 | 0.041893 |
| CERKL | 1.151844 | 0.554984 | -1.05343 | 0.001196 | 0.040614 |
| MAML2 | 1.203716 | 0.41672 | -1.53034 | 0.001349 | 0.041893 |
| PLS1 | 7.290107 | 3.578393 | -1.02663 | 0.001519 | 0.043359 |
| CARMIL1 | 2.05893 | 0.954904 | -1.10847 | 0.001461 | 0.04303 |
| IFI16 | 7.295813 | 2.312146 | -1.65784 | 0.000936 | 0.039132 |
| COL6A3 | 11.52187 | 1.919266 | -2.58575 | 0.001644 | 0.044797 |
| ALOX5 | 2.46398 | 0.746934 | -1.72194 | 0.00158 | 0.043839 |
| GPX8 | 2.68042 | 0.633397 | -2.08128 | 0.000761 | 0.036953 |
| CTTNBP2NL | 1.947663 | 0.816447 | -1.25431 | 0.001996 | 0.047096 |
| EVI2A | 2.252767 | 0.886399 | -1.34567 | 0.000642 | 0.03672 |
| ZDHHC13 | 1.111349 | 0.412014 | -1.43155 | 5.41E-05 | 0.024487 |
| CYP2D6 | 57.56659 | 144.5121 | 1.327887 | 0.00059 | 0.036536 |
| B3GNT9 | 2.017219 | 0.633588 | -1.67075 | 5.98E-05 | 0.024487 |
| ELK3 | 5.886149 | 2.828369 | -1.05735 | 0.001709 | 0.045008 |
| DPYD | 10.08975 | 4.978102 | -1.01922 | 0.000496 | 0.035979 |
| ARNT2 | 1.307791 | 0.589232 | -1.15022 | 0.000899 | 0.038414 |
| LUM | 23.50023 | 3.526925 | -2.73619 | 0.000193 | 0.032229 |
| GRAMD1B | 1.243742 | 0.446438 | -1.47815 | 0.001461 | 0.04303 |
| B3GALNT1 | 1.214065 | 0.503306 | -1.27034 | 0.000232 | 0.032229 |
| CAV2 | 9.216567 | 4.395259 | -1.06828 | 0.001996 | 0.047096 |
| SAMD9L | 1.864907 | 0.655146 | -1.50922 | 0.001709 | 0.045008 |
| LRRC17 | 3.051439 | 0.425548 | -2.8421 | 0.000266 | 0.032898 |
| GNG2 | 1.381071 | 0.67199 | -1.03928 | 0.00067 | 0.03672 |
| PCDH17 | 1.124908 | 0.541666 | -1.05433 | 0.000899 | 0.038414 |
| AMPD3 | 0.752368 | 0.289544 | -1.37766 | 4.88E-05 | 0.024487 |
| TPM1 | 13.85858 | 6.329264 | -1.13067 | 3.76E-05 | 0.024487 |
| CTNND2 | 2.767057 | 1.355077 | -1.02998 | 0.002209 | 0.048713 |
| FPR3 | 5.022199 | 1.627855 | -1.62535 | 0.001059 | 0.039132 |
| DKK4 | 1.522278 | 10.46451 | 2.7812 | 0.000227 | 0.032229 |
| DSE | 0.876994 | 0.217803 | -2.00954 | 0.000862 | 0.037896 |
| SH3RF1 | 3.526086 | 1.360776 | -1.37364 | 0.000417 | 0.035679 |
| APOL4 | 1.660798 | 0.650095 | -1.35315 | 0.001296 | 0.04119 |
| FAM129B | 21.77321 | 9.198292 | -1.24312 | 0.001404 | 0.042505 |
| SEMA5A | 1.973049 | 0.653423 | -1.59434 | 0.000541 | 0.035979 |
| FUT4 | 1.55176 | 0.539217 | -1.52497 | 0.001709 | 0.045008 |
| WIPF1 | 3.790977 | 1.377966 | -1.46003 | 0.000254 | 0.032229 |
| FZD1 | 3.324846 | 0.76289 | -2.12374 | 0.001777 | 0.045161 |
| ELF4 | 2.978977 | 1.089796 | -1.45076 | 0.002326 | 0.048939 |
| EPHA3 | 1.436249 | 0.224037 | -2.68049 | 0.000221 | 0.032229 |
| SKAP1 | 3.539363 | 1.692267 | -1.06453 | 0.002239 | 0.048713 |
| COL3A1 | 111.4715 | 19.71461 | -2.49934 | 0.002239 | 0.048713 |
| CDH11 | 1.511844 | 0.202666 | -2.89914 | 0.001921 | 0.046731 |
| HIF1A | 25.5854 | 8.063364 | -1.66587 | 0.000305 | 0.032915 |
| JAK2 | 2.019123 | 0.705517 | -1.51698 | 0.000221 | 0.032229 |
| RAVER2 | 1.725268 | 0.780124 | -1.14504 | 0.000455 | 0.035679 |
| PTPRC | 3.010306 | 1.080773 | -1.47785 | 0.002239 | 0.048713 |
| SOCS1 | 5.257121 | 2.058733 | -1.35252 | 0.000279 | 0.032898 |
| UNC5B | 3.76686 | 1.26463 | -1.57465 | 0.000761 | 0.036953 |
| MNDA | 2.622066 | 0.76801 | -1.77151 | 0.000109 | 0.030333 |
| PLSCR1 | 9.163421 | 3.419958 | -1.42191 | 6.42E-06 | 0.024487 |
| AKT3 | 1.954685 | 0.504026 | -1.95537 | 0.001519 | 0.043359 |
| FYB1 | 2.168547 | 0.813573 | -1.41438 | 0.00067 | 0.03672 |
| PLXDC2 | 1.831589 | 0.726753 | -1.33356 | 0.001103 | 0.039945 |
| MAP1LC3A | 10.43166 | 23.58842 | 1.177109 | 0.001777 | 0.045161 |
| SMIM24 | 12.57666 | 4.709908 | -1.41698 | 0.001426 | 0.04303 |
| C14orf132 | 1.121578 | 0.536962 | -1.06264 | 0.002326 | 0.048939 |
| COL12A1 | 2.185836 | 0.590498 | -1.88818 | 0.001996 | 0.047096 |
| APOBEC3G | 1.758053 | 0.65447 | -1.42558 | 0.000334 | 0.033367 |
| KDELC2 | 4.802593 | 2.364727 | -1.02214 | 9.86E-05 | 0.030333 |
| RCSD1 | 1.730307 | 0.771521 | -1.16525 | 0.001296 | 0.04119 |
| VASH1 | 2.515865 | 1.083344 | -1.21556 | 0.001921 | 0.046731 |
| CIP2A | 1.850399 | 0.820122 | -1.17393 | 0.001519 | 0.043359 |
| STK17B | 2.512376 | 0.898142 | -1.48404 | 3.76E-05 | 0.024487 |
| AFAP1 | 1.571521 | 0.529469 | -1.56954 | 0.00158 | 0.043839 |
| APOBEC3D | 1.233233 | 0.518531 | -1.24994 | 0.000109 | 0.030333 |
| PDGFRA | 2.552266 | 0.475665 | -2.42376 | 0.000616 | 0.03672 |
| PQLC3 | 7.358392 | 3.481136 | -1.07983 | 0.000399 | 0.034771 |
| CLDN11 | 1.857911 | 0.401692 | -2.20952 | 0.001349 | 0.041893 |
| CXCL5 | 13.94638 | 1.149592 | -3.6007 | 0.000594 | 0.036587 |
| CCNG2 | 2.371389 | 1.113813 | -1.09023 | 0.000232 | 0.032229 |
| SSC5D | 0.849525 | 0.162004 | -2.39063 | 0.000827 | 0.037409 |
| CDC42BPG | 1.348269 | 0.480089 | -1.48973 | 0.002326 | 0.048939 |
| P2RY13 | 0.940765 | 0.360848 | -1.38244 | 0.001709 | 0.045008 |
| BICC1 | 7.438865 | 1.564043 | -2.2498 | 0.001149 | 0.04026 |
| TNFSF10 | 40.8997 | 20.2534 | -1.01393 | 0.000211 | 0.032229 |
| ACSS1 | 2.594829 | 1.174784 | -1.14324 | 0.001777 | 0.045161 |
| OGFRL1 | 1.768593 | 0.858275 | -1.04309 | 0.000541 | 0.035979 |
| CSF2RB | 1.245017 | 0.444569 | -1.48569 | 0.000761 | 0.036953 |
| LXN | 2.995525 | 0.986828 | -1.60194 | 0.000279 | 0.032898 |
| LPAR5 | 0.831461 | 0.259042 | -1.68246 | 0.000221 | 0.032229 |
| LPCAT4 | 2.901547 | 1.441865 | -1.00889 | 0.000729 | 0.036953 |
| WDFY3 | 1.320545 | 0.659495 | -1.0017 | 0.002074 | 0.047802 |
| COL8A2 | 2.173342 | 0.199772 | -3.44349 | 0.000761 | 0.036953 |
| SSPN | 0.857822 | 0.160636 | -2.41689 | 0.00067 | 0.03672 |
| CHSY1 | 5.930738 | 1.842197 | -1.68678 | 0.001709 | 0.045008 |
| VNN2 | 15.00536 | 5.018108 | -1.58026 | 0.002155 | 0.048538 |
| GEM | 4.542247 | 1.123863 | -2.01494 | 0.000334 | 0.033367 |
| ZNF738 | 0.721403 | 0.322727 | -1.16049 | 0.001921 | 0.046731 |
| CYP2S1 | 2.60079 | 0.692044 | -1.91001 | 0.000435 | 0.035679 |
| PMP22 | 7.018806 | 2.15769 | -1.70174 | 0.000827 | 0.037409 |
| DOCK10 | 1.041469 | 0.294337 | -1.82308 | 3.76E-05 | 0.024487 |
| CLEC7A | 1.214537 | 0.365031 | -1.73431 | 0.000729 | 0.036953 |
| HOXB5 | 0.723183 | 0.279645 | -1.37077 | 0.001848 | 0.045984 |
| GLIS3 | 1.97874 | 0.375628 | -2.39721 | 0.000334 | 0.033367 |
| ZFP30 | 0.674917 | 0.329728 | -1.03343 | 0.000365 | 0.034287 |
| MXRA5 | 3.268664 | 0.283301 | -3.52829 | 0.001245 | 0.041129 |
| SLC9A9 | 1.756815 | 0.780839 | -1.16987 | 0.000699 | 0.036953 |
| LIMA1 | 6.478702 | 2.776556 | -1.22241 | 0.000761 | 0.036953 |
| LCP2 | 2.74366 | 1.116449 | -1.29718 | 0.000729 | 0.036953 |
| GPR34 | 2.303424 | 0.828227 | -1.47568 | 0.000793 | 0.036953 |
| EDIL3 | 3.231729 | 0.76705 | -2.07491 | 0.001848 | 0.045984 |
| HEG1 | 4.776946 | 1.776998 | -1.42665 | 0.000793 | 0.036953 |
| ITPRIPL2 | 3.36458 | 1.470023 | -1.19459 | 0.000114 | 0.030333 |
| CRMP1 | 1.444184 | 0.647455 | -1.1574 | 0.002239 | 0.048713 |
| TNFSF13B | 2.448598 | 0.774928 | -1.65982 | 0.000862 | 0.037896 |
| NUAK1 | 3.86008 | 1.68412 | -1.19664 | 0.000793 | 0.036953 |
| RASA3 | 3.056737 | 1.388063 | -1.13892 | 0.000221 | 0.032229 |
| MFSD6 | 3.39292 | 1.154626 | -1.5551 | 6.96E-05 | 0.024487 |
| LARP6 | 1.743815 | 0.50552 | -1.78641 | 0.000899 | 0.038414 |
| PMAIP1 | 0.81438 | 0.260235 | -1.64588 | 0.000455 | 0.035679 |
| RAB8B | 5.590717 | 2.567527 | -1.12265 | 0.000103 | 0.030333 |
| SLC25A24 | 2.206599 | 0.595812 | -1.8889 | 0.000541 | 0.035979 |
| GLS | 6.095915 | 2.724661 | -1.16177 | 0.000193 | 0.032229 |
| MICB | 2.61889 | 1.169937 | -1.16252 | 0.000211 | 0.032229 |
| EVC | 1.586371 | 0.582433 | -1.44556 | 0.001519 | 0.043359 |
| FCGR2A | 4.555554 | 1.733565 | -1.39388 | 0.002326 | 0.048939 |
| MSRB3 | 2.07272 | 0.875332 | -1.24362 | 0.000349 | 0.033367 |
| MTCL1 | 0.950072 | 0.288748 | -1.71823 | 0.000616 | 0.03672 |
| IQGAP1 | 6.150866 | 2.276251 | -1.43413 | 0.000455 | 0.035679 |
| SULF1 | 3.009867 | 0.685982 | -2.13346 | 0.002326 | 0.048939 |
| SPINT2 | 12.6636 | 4.789985 | -1.40259 | 0.000232 | 0.032229 |
| SAMD9 | 1.170076 | 0.525194 | -1.15568 | 0.001296 | 0.04119 |
| LIFR | 1.447839 | 0.46061 | -1.65228 | 0.001149 | 0.04026 |
| SFXN3 | 4.351188 | 2.112588 | -1.0424 | 0.000232 | 0.032229 |
| SEMA6A | 2.33913 | 0.859267 | -1.44479 | 0.001996 | 0.047096 |
| NCOA7 | 5.887125 | 2.581824 | -1.18917 | 8.09E-05 | 0.027557 |
| PTGS1 | 1.433817 | 0.566625 | -1.33939 | 0.001296 | 0.04119 |
| SPIN4 | 1.225693 | 0.458504 | -1.41859 | 0.000381 | 0.034364 |
| RUNX1 | 1.835239 | 0.841677 | -1.12463 | 0.000541 | 0.035979 |
| IRF8 | 3.757402 | 1.587177 | -1.24327 | 0.001461 | 0.04303 |
| MCUB | 2.205189 | 0.76999 | -1.51799 | 0.000899 | 0.038414 |
| FILIP1L | 2.922717 | 1.306637 | -1.16145 | 0.000496 | 0.035979 |
| TMEM159 | 2.337868 | 0.853971 | -1.45293 | 0.000346 | 0.033367 |
| UBASH3B | 0.919534 | 0.219502 | -2.06667 | 0.000455 | 0.035679 |
| CXCL6 | 8.487939 | 0.893013 | -3.24866 | 0.001205 | 0.040792 |
| SCD5 | 6.066922 | 0.266955 | -4.5063 | 0.00067 | 0.03672 |
| GUCY1A1 | 2.009507 | 0.568706 | -1.82109 | 0.000976 | 0.039132 |
| CTSS | 33.00713 | 14.03728 | -1.23351 | 0.001709 | 0.045008 |
| GUCY1B1 | 3.392348 | 1.126934 | -1.58988 | 0.000145 | 0.032229 |
| BACE2 | 7.269501 | 1.874302 | -1.9555 | 0.000518 | 0.035979 |
| RBMS2 | 1.606622 | 0.734019 | -1.13014 | 0.000221 | 0.032229 |
| DAB2 | 10.96725 | 5.239057 | -1.06582 | 0.001461 | 0.04303 |
| TPBG | 1.389082 | 0.339816 | -2.03131 | 0.001149 | 0.04026 |
| ZEB2 | 0.846111 | 0.323521 | -1.38699 | 0.000349 | 0.033367 |
| CERCAM | 2.803271 | 1.094695 | -1.35658 | 0.001059 | 0.039132 |
| CDCP1 | 2.143911 | 0.476446 | -2.16986 | 0.002239 | 0.048713 |
| SLC12A8 | 3.89285 | 1.657687 | -1.23165 | 3.97E-05 | 0.024487 |
| NCEH1 | 5.903158 | 1.138615 | -2.37421 | 0.000334 | 0.033367 |
| SLIT2 | 1.026225 | 0.129153 | -2.9902 | 0.000349 | 0.033367 |
| GALNT7 | 1.020611 | 0.364323 | -1.48614 | 0.000518 | 0.035979 |
| DSEL | 0.82283 | 0.343481 | -1.26036 | 0.00158 | 0.043839 |
| GAL3ST4 | 1.929388 | 0.442947 | -2.12294 | 0.000435 | 0.035679 |
| GPRC5B | 4.890029 | 1.478517 | -1.72569 | 6.62E-05 | 0.024487 |
| TWSG1 | 3.766396 | 1.491743 | -1.33619 | 9.08E-06 | 0.024487 |
| CYBB | 7.980798 | 2.823841 | -1.49887 | 0.001848 | 0.045984 |
| PF4V1 | 1.330224 | 0.272255 | -2.28864 | 0.000166 | 0.032229 |
| FRMD6 | 1.499773 | 0.529236 | -1.50276 | 6.30E-05 | 0.024487 |
| NEO1 | 5.871349 | 2.400561 | -1.29032 | 0.000291 | 0.032898 |
| ANXA4 | 24.43824 | 9.801698 | -1.31804 | 3.22E-05 | 0.024487 |
| TMEM229B | 0.973673 | 0.404209 | -1.26834 | 0.000793 | 0.036953 |
| TLR5 | 1.374834 | 0.570141 | -1.26987 | 0.001461 | 0.04303 |
| SERPINH1 | 41.8667 | 20.67075 | -1.01821 | 0.000729 | 0.036953 |
| DSG2 | 13.14799 | 4.66355 | -1.49534 | 0.001996 | 0.047096 |
| SPRED1 | 2.761603 | 1.018984 | -1.43838 | 0.000381 | 0.034364 |
| BTC | 0.899694 | 0.275299 | -1.70844 | 0.000293 | 0.032898 |
| MATN2 | 6.334611 | 2.709439 | -1.22526 | 0.00059 | 0.036536 |
| THBS2 | 9.780088 | 2.367714 | -2.04635 | 0.001059 | 0.039132 |
| CSF1 | 11.1265 | 5.071621 | -1.13348 | 0.000936 | 0.039132 |
| ADGRE5 | 10.32586 | 5.148998 | -1.0039 | 0.000475 | 0.035979 |
| TPM4 | 35.10321 | 12.93231 | -1.44062 | 0.000455 | 0.035679 |
| BIRC3 | 10.87311 | 4.175146 | -1.38087 | 0.002155 | 0.048538 |
| EPHB6 | 4.162635 | 0.715833 | -2.5398 | 0.000793 | 0.036953 |
| A4GNT | 1.237146 | 0.083386 | -3.89106 | 0.001545 | 0.043839 |
| NOTCH2 | 5.783135 | 2.475436 | -1.22417 | 0.001296 | 0.04119 |
| VCAM1 | 9.528739 | 3.063975 | -1.63688 | 0.000349 | 0.033367 |
| MUC1 | 2.890112 | 0.487802 | -2.56676 | 0.001519 | 0.043359 |
| DOCK8 | 1.149965 | 0.443 | -1.37621 | 2.21E-05 | 0.024487 |
| FAM102B | 1.562236 | 0.601998 | -1.37578 | 4.00E-06 | 0.024487 |
| PMEPA1 | 12.35888 | 1.403839 | -3.1381 | 0.001245 | 0.041129 |
| CAV1 | 12.66414 | 6.003931 | -1.07677 | 0.00158 | 0.043839 |
| ARID5B | 2.930456 | 1.444653 | -1.0204 | 0.001296 | 0.04119 |
| MTHFD2 | 2.061566 | 0.885888 | -1.21854 | 0.001196 | 0.040614 |
| SLC7A7 | 3.348882 | 1.289505 | -1.37686 | 0.001059 | 0.039132 |
| FMNL3 | 1.846977 | 0.805816 | -1.19664 | 0.00158 | 0.043839 |
| PPP1R18 | 13.58153 | 6.326561 | -1.10215 | 0.000232 | 0.032229 |
| ARAP2 | 0.909972 | 0.430941 | -1.07833 | 0.001996 | 0.047096 |
| HRH1 | 0.861446 | 0.18514 | -2.21814 | 0.001149 | 0.04026 |
| OLFML1 | 3.401037 | 1.334524 | -1.34965 | 0.000279 | 0.032898 |
| CHST11 | 3.235512 | 1.160887 | -1.47877 | 0.00067 | 0.03672 |
| LTBP2 | 4.189425 | 1.31401 | -1.67278 | 0.001921 | 0.046731 |
| ELOVL7 | 3.843043 | 1.157211 | -1.7316 | 0.001017 | 0.039132 |
| ZNF14 | 1.284797 | 0.616313 | -1.05981 | 0.000899 | 0.038414 |
| SPINT1 | 11.15416 | 2.298984 | -2.27851 | 0.000334 | 0.033367 |
| TGFA | 1.99213 | 0.539466 | -1.88471 | 0.000475 | 0.035979 |
| PTPRE | 1.124499 | 0.387144 | -1.53834 | 0.002074 | 0.047802 |
| SYK | 2.691157 | 0.977041 | -1.46174 | 0.000976 | 0.039132 |
| KCTD12 | 5.75338 | 1.912032 | -1.5893 | 0.001644 | 0.044797 |
| CGAS | 1.088774 | 0.367361 | -1.56743 | 5.98E-05 | 0.024487 |
| PAPLN | 2.3318 | 1.015284 | -1.19956 | 0.002326 | 0.048939 |
| RAB23 | 0.866265 | 0.365743 | -1.24398 | 0.000761 | 0.036953 |
| CD3G | 0.844327 | 0.230516 | -1.87294 | 0.002326 | 0.048939 |
