# Supplementary material for: A novel panel based on immune infiltration and tumor mutational burden for prognostic prediction in hepatocellular carcinoma
Source: Aging (Albany NY). 2021 Mar 10;13(6):8563–87. doi: 10.18632/aging.202670 (PMC8034943; doi:10.18632/aging.202670)
Supplement: Supplementary Table 4 [file aging-13-202670-s005.docx]

**Supplementary Table 4. Genes associated with different immune cells and modules**

| **Supplementary Table 4.1 Genes associated with** **InfiltrationScore in blue module** | | | | | |
| --- | --- | --- | --- | --- | --- |
| probes | moduleColor | GS.InfiltrationScore | p.GS.InfiltrationScore | MMblue | p.MMblue |
| ST8SIA4 | blue | 0.6250 | 0.0000 | 0.9026 | 0.0000 |
| HLA-DRA | blue | 0.6487 | 0.0000 | 0.8290 | 0.0000 |
| EVI2A | blue | 0.7033 | 0.0000 | 0.9487 | 0.0000 |
| FPR3 | blue | 0.6384 | 0.0000 | 0.9234 | 0.0000 |
| PTPRC | blue | 0.4634 | 0.0001 | 0.8909 | 0.0000 |
| MNDA | blue | 0.6760 | 0.0000 | 0.9108 | 0.0000 |
| FYB1 | blue | 0.5718 | 0.0000 | 0.9408 | 0.0000 |
| RCSD1 | blue | 0.5171 | 0.0000 | 0.8178 | 0.0000 |
| P2RY13 | blue | 0.5867 | 0.0000 | 0.8537 | 0.0000 |
| CSF2RB | blue | 0.6189 | 0.0000 | 0.9280 | 0.0000 |
| CLEC7A | blue | 0.6387 | 0.0000 | 0.9316 | 0.0000 |
| LCP2 | blue | 0.6932 | 0.0000 | 0.9802 | 0.0000 |
| CYBB | blue | 0.6756 | 0.0000 | 0.9242 | 0.0000 |
| DOCK8 | blue | 0.5129 | 0.0000 | 0.8461 | 0.0000 |
|  |  |  |  |  |  |
| **Supplementary Table 4.2 Genes associated with Macrophage in blue module** | | | | | |
| probes | moduleColor | GS.Macrophage | p.GS.Macrophage | MMblue | p.MMblue |
| ST8SIA4 | blue | 0.5773 | 0.0000 | 0.9026 | 0.0000 |
| HLA-DRA | blue | 0.5986 | 0.0000 | 0.8290 | 0.0000 |
| EVI2A | blue | 0.6280 | 0.0000 | 0.9487 | 0.0000 |
| FPR3 | blue | 0.6361 | 0.0000 | 0.9234 | 0.0000 |
| PTPRC | blue | 0.4103 | 0.0004 | 0.8909 | 0.0000 |
| MNDA | blue | 0.5732 | 0.0000 | 0.9108 | 0.0000 |
| FYB1 | blue | 0.5196 | 0.0000 | 0.9408 | 0.0000 |
| RCSD1 | blue | 0.5261 | 0.0000 | 0.8178 | 0.0000 |
| P2RY13 | blue | 0.6665 | 0.0000 | 0.8537 | 0.0000 |
| CSF2RB | blue | 0.5444 | 0.0000 | 0.9280 | 0.0000 |
| CLEC7A | blue | 0.5836 | 0.0000 | 0.9316 | 0.0000 |
| LCP2 | blue | 0.6347 | 0.0000 | 0.9802 | 0.0000 |
| CYBB | blue | 0.6614 | 0.0000 | 0.9242 | 0.0000 |
|  |  |  |  |  |  |
| **Supplementary Table 4.3 Genes associated with DC in blue module** | | | | |  |
| probes | moduleColor | GS.DC | p.GS.DC | MMblue | p.MMblue |
| ST8SIA4 | blue | 0.5502 | 0.0000 | 0.9026 | 0.0000 |
| HLA-DRA | blue | 0.5545 | 0.0000 | 0.8290 | 0.0000 |
| EVI2A | blue | 0.6311 | 0.0000 | 0.9487 | 0.0000 |
| FPR3 | blue | 0.6322 | 0.0000 | 0.9234 | 0.0000 |
| PTPRC | blue | 0.4519 | 0.0001 | 0.8909 | 0.0000 |
| MNDA | blue | 0.6526 | 0.0000 | 0.9108 | 0.0000 |
| FYB1 | blue | 0.5500 | 0.0000 | 0.9408 | 0.0000 |
| P2RY13 | blue | 0.4652 | 0.0000 | 0.8537 | 0.0000 |
| CSF2RB | blue | 0.5822 | 0.0000 | 0.9280 | 0.0000 |
| CLEC7A | blue | 0.6122 | 0.0000 | 0.9316 | 0.0000 |
| LCP2 | blue | 0.6040 | 0.0000 | 0.9802 | 0.0000 |
| CYBB | blue | 0.6647 | 0.0000 | 0.9242 | 0.0000 |
| DOCK8 | blue | 0.4298 | 0.0002 | 0.8461 | 0.0000 |
|  |  |  |  |  |  |
| **Supplementary Table 4.4 Genes associated with MAIT in blue module** | | | | |  |
| probes | moduleColor | GS.MAIT | p.GS.MAIT | MMblue | p.MMblue |
| ST8SIA4 | blue | 0.5509 | 0.0000 | 0.9026 | 0.0000 |
| HLA-DRA | blue | 0.6264 | 0.0000 | 0.8290 | 0.0000 |
| EVI2A | blue | 0.6274 | 0.0000 | 0.9487 | 0.0000 |
| FPR3 | blue | 0.5012 | 0.0000 | 0.9234 | 0.0000 |
| PTPRC | blue | 0.4625 | 0.0001 | 0.8909 | 0.0000 |
| MNDA | blue | 0.4724 | 0.0000 | 0.9108 | 0.0000 |
| FYB1 | blue | 0.5114 | 0.0000 | 0.9408 | 0.0000 |
| RCSD1 | blue | 0.5851 | 0.0000 | 0.8178 | 0.0000 |
| P2RY13 | blue | 0.5785 | 0.0000 | 0.8537 | 0.0000 |
| CSF2RB | blue | 0.5155 | 0.0000 | 0.9280 | 0.0000 |
| CLEC7A | blue | 0.4853 | 0.0000 | 0.9316 | 0.0000 |
| LCP2 | blue | 0.6008 | 0.0000 | 0.9802 | 0.0000 |
| CYBB | blue | 0.4832 | 0.0000 | 0.9242 | 0.0000 |
| DOCK8 | blue | 0.4215 | 0.0003 | 0.8461 | 0.0000 |
|  |  |  |  |  |  |
| **Supplementary Table 4.5 Genes associated with Th17 in blue module** | | | | |  |
| probes | moduleColor | GS.Th17 | p.GS.Th17 | MMblue | p.MMblue |
| ST8SIA4 | blue | 0.5457 | 0.0000 | 0.9026 | 0.0000 |
| HLA-DRA | blue | 0.4335 | 0.0002 | 0.8290 | 0.0000 |
| EVI2A | blue | 0.5128 | 0.0000 | 0.9487 | 0.0000 |
| FPR3 | blue | 0.4115 | 0.0004 | 0.9234 | 0.0000 |
| MNDA | blue | 0.5209 | 0.0000 | 0.9108 | 0.0000 |
| FYB1 | blue | 0.4358 | 0.0002 | 0.9408 | 0.0000 |
| CSF2RB | blue | 0.4545 | 0.0001 | 0.9280 | 0.0000 |
| CLEC7A | blue | 0.4478 | 0.0001 | 0.9316 | 0.0000 |
| LCP2 | blue | 0.5199 | 0.0000 | 0.9802 | 0.0000 |
| CYBB | blue | 0.4344 | 0.0002 | 0.9242 | 0.0000 |
| DOCK8 | blue | 0.4549 | 0.0001 | 0.8461 | 0.0000 |
|  |  |  |  |  |  |
| **Supplementary Table 4.6 Genes associated with DC in pink module** | | | | |  |
| probes | moduleColor | GS.DC | p.GS.DC | MMpink | p.MMpink |
| SLC9A9 | pink | 0.4599 | 0.0001 | 0.8500 | 0.0000 |
| TNFSF13B | pink | 0.5152 | 0.0000 | 0.9010 | 0.0000 |
|  |  |  |  |  |  |
| **Supplementary Table 4.7 Genes associated with DC in magenta module** | | | | |  |
| probes | moduleColor | GS.DC | p.GS.DC | MMmagenta | p.MMmagenta |
| CXCL5 | magenta | 0.4041 | 0.0005 | 0.8753 | 0.0000 |
| PTGS1 | magenta | 0.4896 | 0.0000 | 0.8825 | 0.0000 |
|  |  |  |  |  |  |
| **Supplementary Table 4.8 Genes associated with MAIT in red module** | | | | |  |
| probes | moduleColor | GS.MAIT | p.GS.MAIT | MMred | p.MMred |
| LUM | red | 0.4074 | 0.0005 | 0.9469 | 0.0000 |
| DSE | red | 0.4993 | 0.0000 | 0.9240 | 0.0000 |
| LXN | red | 0.4369 | 0.0002 | 0.9538 | 0.0000 |
| GEM | red | 0.4777 | 0.0000 | 0.9059 | 0.0000 |
| ZEB2 | red | 0.4913 | 0.0000 | 0.9379 | 0.0000 |
| HRH1 | red | 0.4387 | 0.0001 | 0.8268 | 0.0000 |
| KCTD12 | red | 0.5557 | 0.0000 | 0.9650 | 0.0000 |
|  |  |  |  |  |  |
| **Supplementary Table 4.9 Genes associated with Macrophage in red module** | | | | | |
| probes | moduleColor | GS.Macrophage | p.GS.Macrophage | MMred | p.MMred |
| DSE | red | 0.5230 | 0.0000 | 0.9240 | 0.0000 |
| ZEB2 | red | 0.4550 | 0.0001 | 0.9379 | 0.0000 |
| HRH1 | red | 0.4423 | 0.0001 | 0.8268 | 0.0000 |
| KCTD12 | red | 0.5140 | 0.0000 | 0.9650 | 0.0000 |
|  |  |  |  |  |  |

| **Supplementary Table 4.10 Genes associated with MAIT in green module** | | | | |  |
| --- | --- | --- | --- | --- | --- |
| probes | moduleColor | GS.MAIT | p.GS.MAIT | MMgreen | p.MMgreen |
| DCN | green | 0.4882 | 0.0000 | 0.8218 | 0.0000 |
| GPX8 | green | 0.4079 | 0.0005 | 0.8826 | 0.0000 |
| GNG2 | green | 0.5632 | 0.0000 | 0.8261 | 0.0000 |
| PMP22 | green | 0.4320 | 0.0002 | 0.9180 | 0.0000 |
| THBS2 | green | 0.4507 | 0.0001 | 0.9247 | 0.0000 |
| FMNL3 | green | 0.4284 | 0.0002 | 0.8979 | 0.0000 |
